# Electronic Excited States from Physically Constrained Machine Learning

**DOI:** 10.1021/acscentsci.3c01480

**Published:** 2024-02-29

**Authors:** Edoardo Cignoni, Divya Suman, Jigyasa Nigam, Lorenzo Cupellini, Benedetta Mennucci, Michele Ceriotti

**Affiliations:** † Dipartimento di Chimica e Chimica Industriale, Università di Pisa, 56126 Pisa, Italy; ‡ Laboratory of Computational Science and Modeling, Institut des Matériaux, École Polytechnique Fédérale de Lausanne, 1015 Lausanne, Switzerland; § Division of Chemistry and Chemical Engineering, California Institute of Technology, Pasadena, California 91125, United States

## Abstract

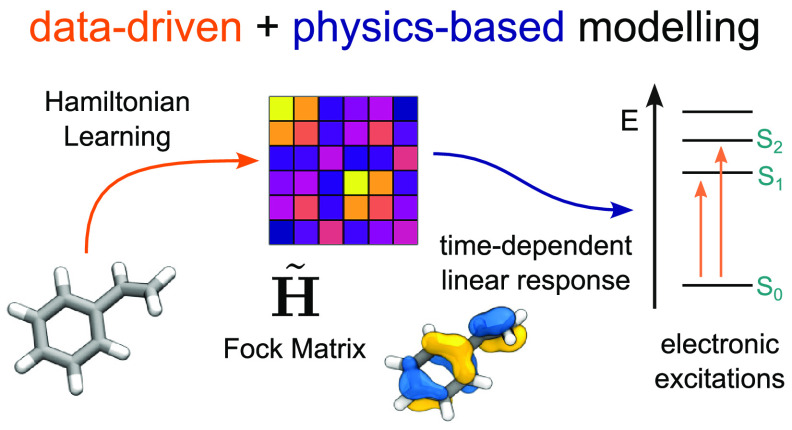

Data-driven techniques are increasingly used to replace electronic-structure calculations of matter. In this context, a relevant question is whether machine learning (ML) should be applied directly to predict the desired properties or combined explicitly with physically grounded operations. We present an example of an integrated modeling approach in which a symmetry-adapted ML model of an effective Hamiltonian is trained to reproduce electronic excitations from a quantum-mechanical calculation. The resulting model can make predictions for molecules that are much larger and more complex than those on which it is trained and allows for dramatic computational savings by indirectly targeting the outputs of well-converged calculations while using a parametrization corresponding to a minimal atom-centered basis. These results emphasize the merits of intertwining data-driven techniques with physical approximations, improving the transferability and interpretability of ML models without affecting their accuracy and computational efficiency and providing a blueprint for developing ML-augmented electronic-structure methods.

## Abstract 2

Development of a hybrid model integrating a data-driven prediction of molecular Hamiltonians and physics-based postprocessing yields an accurate and balanced description of excited states.

## Introduction

Machine learning (ML) methods have been applied very successfully to circumvent the complexity and computational cost of physics-based modeling.^1,2^ For example, ML interatomic potentials trained on quantum mechanical (QM) calculations have become ubiquitous, making it possible to simulate the structure and stability of complex systems in realistic thermodynamic conditions.^3−5^ ML approaches are increasingly applied to a broader array of quantum mechanical properties,^6^ from the ground-state electron density^7−9^ to electronic excitations,^10−14^ the latter of which are the main focus of the present study. In QM calculations, these properties are often the result of a sequence of computational steps that operate on intermediate quantities describing the electronic structure of a molecule. For instance, mean-field methods such as Hartree–Fock and Kohn–Sham density functional theory (DFT)^15,16^ evaluate a self-consistent, effective single-particle Hamiltonian, which can be diagonalized to obtain numerous properties of the system. Methods based on this single-particle picture also form the basis of more accurate *ab initio* methods such as complete active space (CAS) or coupled-cluster (CC) theories, as well as of less demanding semiempirical methods,^17−19^ which parametrize the Hamiltonian using *ab initio* and empirical data. In addition to the parametrization, which avoids computing many expensive integrals, semiempirical schemes usually work in a “minimal basis”^20,21^ and consider only valence electrons, discarding core electrons and high-energy virtual orbitals,^22^ to further speed up the calculations. Significant work has been devoted to exploring these approximations, which can be used as inspiration to design ML schemes for electronic properties.^23^

When the single-particle wave function is expressed in terms of an atom-centered basis, the elements of the Hamiltonian matrix are indexed by two atoms and determined by interactions with their neighbors, which makes them very well suited as the target of an ML model built on geometric and chemical information.^24^ Over the past few years, several works have discussed the prediction of single-particle electronic Hamiltonians of molecular systems.^25−30^ The electronic structure is constrained by physical symmetries, which can be exploited by constructing equivariant ML schemes that ensure that the data-driven model conforms to these constraints.^24,31,32^ Once the Hamiltonian matrix is obtained, all sorts of ground-state properties, such as the electron density, can be obtained with simple, inexpensive manipulations. Furthermore, excited states can also be predicted, at least approximately, by postprocessing the ground-state single-particle Hamiltonian. As a matter of fact, an ML algorithm could be built to directly target the property one wants to predict, e.g., electronic excitation energies^10,33,34^ or HOMO–LUMO gaps.^35^ Here, we pursue an alternative strategy, which integrates data-driven modeling and QM calculations more closely.

We build a symmetry-adapted ML model with an intermediate layer that mimics a minimal-basis, single-particle electronic Hamiltonian, which is then used to compute molecular orbital (MO) energy levels and atomic charges. We then train this model against the MOs obtained from quantum chemical calculations with a richer basis set. The resulting architecture inherits the accuracy of these more refined QM calculations as well as the transferability to larger, more complex molecules while being orders of magnitude faster. This approach also enables predictions of molecular excited states, which we demonstrate using the simplified Tamm–Dancoff Approximation (sTDA)^36,37^ to calculate valence excitation energies.

We analyze the resulting model for a data set of hydrocarbons, training on a few small molecules and assessing predictions on much larger systems. Our model cannot only reproduce the energies and shapes of MOs of previously unseen molecules but also can predict their excitation energies with remarkable accuracy. We finally showcase an example of calculating the vibronic spectra of a molecule not present in the training set. Our observations have relevance beyond the specific type of excited-state calculations we apply, as they indicate that models combining data-driven steps with physically motivated manipulations and constraints combine the advantages of both approaches and deserve to be more widely adopted as a tool for accurate and affordable atomistic modeling of the electronic properties of molecules and materials.

## Results

The details of our framework are described in the Methods section and in the Supporting Information. However, to better appreciate the results, we outline the motivation behind some of our technical choices. Our overarching goal is to demonstrate how a hybrid ML scheme can achieve transferability on different axes. On one axis, we aim to show that it can be trained on small, simple molecules and reach useful accuracy when making predictions on more complicated, larger compounds. On the other axis, we want to demonstrate that the model can predict quantities other than those that the model has been trained on: specifically, electronic excitations and their coupling with nuclear vibrations. With these goals in mind, we train and validate our model on small hydrocarbon systems (specifically ethane, ethene, butadiene, hexane, hexatriene, styrene and isoprene), which provide a concise but representative palette of saturated, unsaturated, and aromatic motifs. A dataset comprising these structures was generated from high-temperature replica-exchange molecular dynamics (REMD) simulations and contains multiple conformers and distorted configurations. We then use the trained model for predictions on larger and more complex molecules, from azulene to beta-carotene. We select classes of compounds with interesting yet well-understood physical effects (e.g., the dependence of band gap on the extent of a conjugated system) and use a simple approximation to compute electronic excitations, allowing for a comparison with explicit quantum mechanical calculations for large molecules and more subtle properties, such as vibronic spectra. Even though our architecture is fully compatible with any deep-learning scheme, we use an equivariant model based on linear regression to emphasize the impact of the coupling between the ML scheme and the physical approximations over the fine-tuning of the architecture of a nonlinear ML model. We finally stress that our methodology is by no means limited to just hydrocarbons and can be extended straightforwardly to more diverse data sets.

### Hybrid ML Architecture

Most of the ML frameworks that predict the Hamiltonian directly target the elements of a single-particle electronic matrix **H** (the Fock or Kohn–Sham matrix) obtained from a quantum mechanical calculation,^24,28,31,32^ which describes the interactions between a suitable set of basis functions centered on the different atoms. The molecular orbitals (MO) and their energies {ε_*n*_} are obtained by diagonalizing the predicted (or quantum mechanical) **H**. A notable exception is given by the SchNet+H approach,^38^ in which the target is a “pseudo-Hamiltonian” **H̃** that is invariant to the molecular orientation, which is diagonalized to obtain eigenvalues {ε̃_*n*_} that are then compared with the reference calculation. This was shown to provide better accuracy (and a much-simplified architecture) than directly targeting the matrix elements, at the cost of losing the natural symmetries of the physical Hamiltonian. Ref (24) also discusses the construction of a symmetry-adapted projected Hamiltonian that reproduces the MO energies from a converged calculation using a smaller set of orbitals and was then used as the target of the ML model. This simplifies the calculation (the cost of diagonalizing a matrix scales cubically with the number of orbitals) but introduces a nonphysical training target, as there is no unique definition of the reduced matrix.

**Figure 1:**
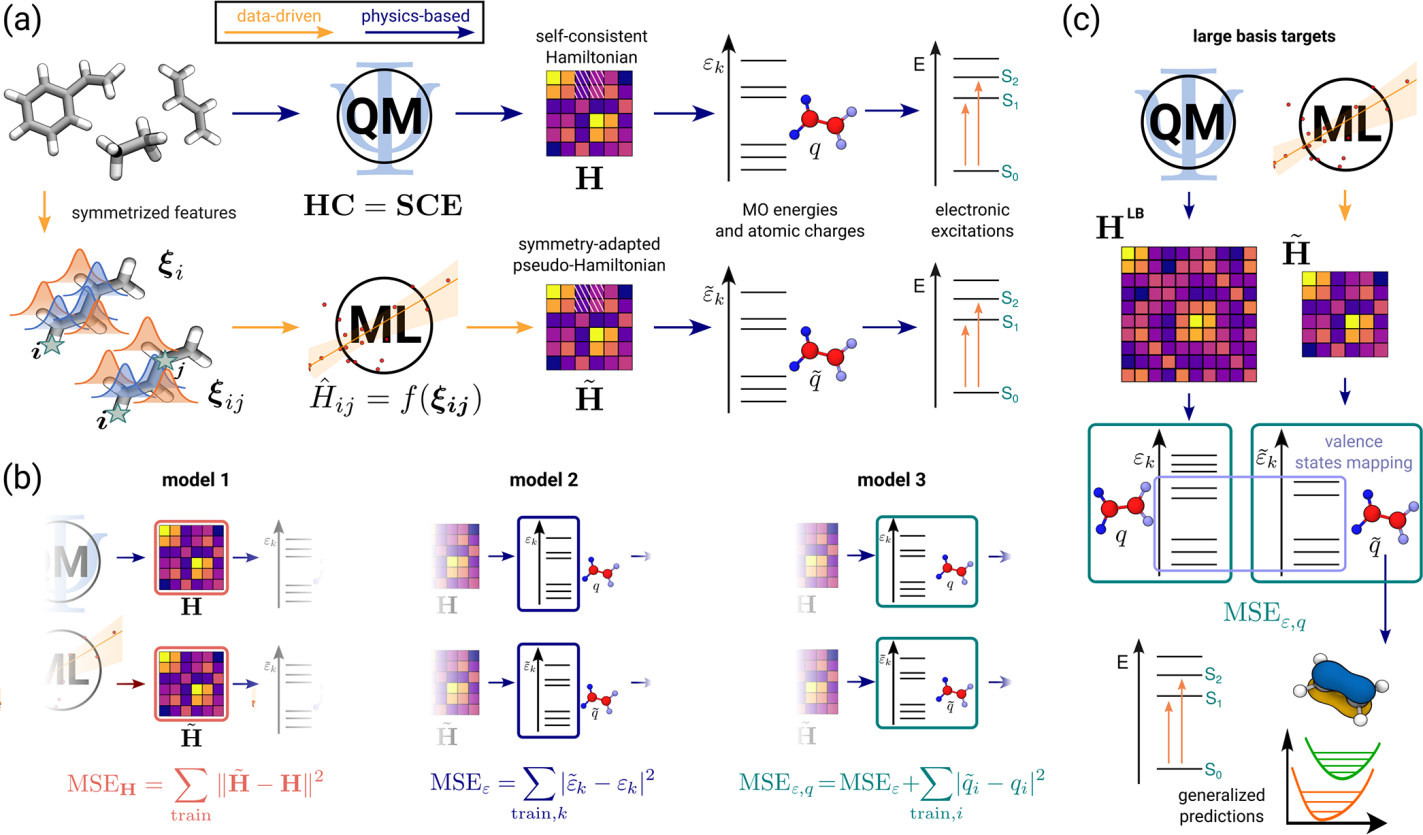
Schematic representation of an indirect learning framework for electronic excitations. (a) An ML model designed to target the molecular orbital (MO) energies ε_*k*_ and the Löwdin charges *q* by using an equivariant model of the single-particle Hamiltonian as an intermediate layer. The Hamiltonian blocks for each pair of atoms (*i*, *j*) in the structure are modeled by learnable functions *f* with corresponding input features ξ. Arrows are color-coded to indicate data-driven predictions and physics-based approximations. (b) We compare different training protocols, targeting the elements of the Hamiltonian (model 1), the minimal-basis eigenvalues (model 2), and both the eigenvalues and Löwdin charges (model 3). (c) Illustration of a model that is trained on charges and a selection of MO energies computed with a larger basis (LBT). Once the effective Hamiltonian is learned, it can be used to compute other electronic-structure quantities, beyond those used during training.

The hybrid architecture we propose here is shown schematically in Figure 1, and it combines the most desirable features of these earlier schemes. We use a symmetry-adapted ridge regression model based on equivariant two-center–one-neighbor atom-density correlation features^24^ to parametrize the matrix elements of an effective minimal-basis Hamiltonian **H̃**. This matrix has a structure and the O(3) symmetries corresponding to an atom-centered STO-3G basis, containing 1s orbitals for H and 1s, 2s, and 2p for C atoms. **H̃** can be used in different ways, corresponding to different strategies to incorporate data-driven techniques in an electronic structure calculation and to the different models and loss functions depicted in Figure 1b.

In model 1, we take the effective Hamiltonian matrix as a literal prediction of the results of a self-consistent STO-3G calculation and compute the loss as the *l*^2^ norm of the difference between the predicted **H̃** and the target **H**. In model 2, we compute the eigenvalues ε̃_*n*_ by diagonalizing **H̃** and define the loss as the mean squared error in reproducing the eigenvalues ε_*n*_ of the STO-3G calculation. In this case, **H̃** plays the role of a “pseudo-Hamiltonian” that (in contrast to SchNet+H) has the correct symmetry properties but is not bound to be equal to the matrix elements computed in a specified minimal basis. The imposed symmetry properties help the model to recover the correct nodal structure of MOs.^24^ Finally, in model 3 we supplement the MO energies with other quantities computed from the electronic-structure calculations and compute a combined loss that measures the errors in reproducing all of the physical constraints. In our case, we use the Löwdin atomic charges from the QM STO-3G calculation. This constraint is meant to guide the model toward a physically grounded prediction of the QM density on each atom. We stress that, in all cases but model 1, minimizing the model loss is a nonconvex optimization problem despite the fact that we use a linear expression for the relation between the matrix elements of **H̃** and the structural descriptors.

**Figure 2:**
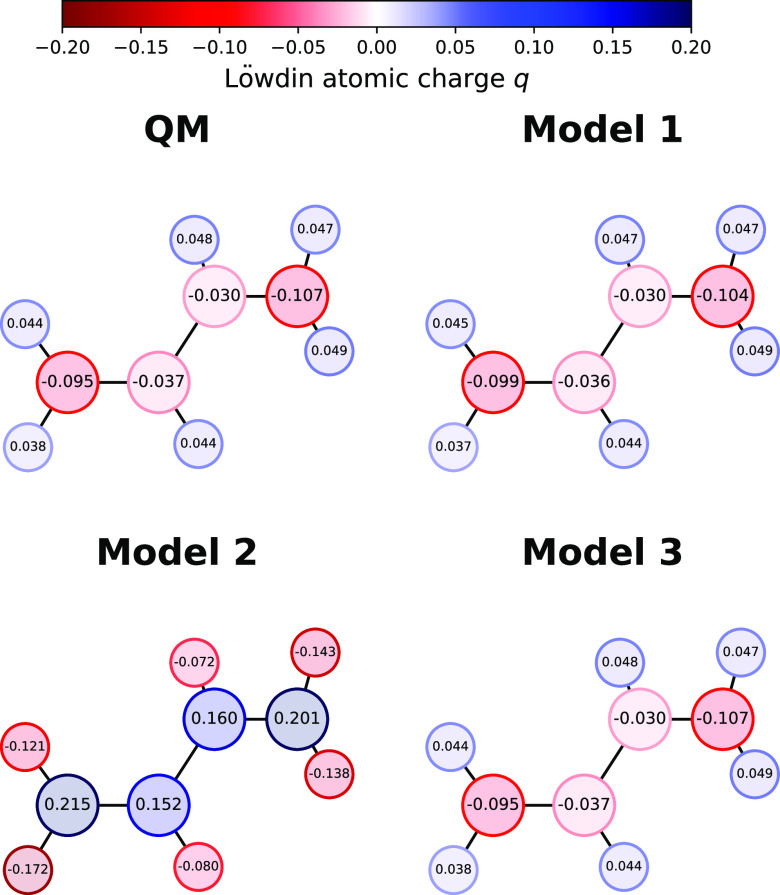
Comparison of atomic Löwdin charges for the three ML models. The QM target is computed at the B3LYP/STO-3G level of theory on a randomly selected geometry of butadiene. Model 1 is trained directly on the STO-3G Hamiltonian. Model 2 is trained indirectly using only MO energies as a target. Model 3 is trained indirectly using MO energies and Löwdin charges.

As shown in Table 1, targeting the matrix elements of the Hamiltonian in a direct learning setup (model 1) leads to consistently larger prediction errors for the single-particle energy levels than using the ML model of the Hamiltonian as an intermediate step in the calculation of the eigenvalues (model 2). However, the indirect optimization leads to dramatic errors in other quantities that can be computed from the Hamiltonian matrix. Model 2 gives unphysical predictions for atomic charges, such as negative charges on hydrogen atoms (Figure 2). Combining multiple targets (model 3) achieves a better-balanced model that improves upon direct Hamiltonian learning for both eigenvalues and atomic charges. The MO energies have slightly larger errors (Table 1), as the composite loss forces the model to both reproduce the MO energies and the overall electronic density (via the atomic charges). Model 3, thus, more faithfully respects the underlying physics, which helps when generalizing to new properties and molecules, as we show in the following sections.

**Table 1:** Performance Comparison of the Three Different ML Models^a^

	= MSE_H_		= MSE_ε_		= MSE_ε,*q*_	
molecule	MAE_ε_ (meV)	MAE_*q*_ (e)	MAE_ε_ (meV)	MAE_*q*_(e)	MAE_ε_ (meV)	MAE_*q*_ (e)
ethane	41.82	1.4 × 10^–3^	9.96	0.11	16.15	4.1 × 10^–4^
ethene	68.45	1.6 × 10^–3^	7.99	0.15	12.37	4.9 × 10^–4^
butadiene	76.92	4.8 × 10^–3^	32.60	0.13	44.50	1.0 × 10^–3^
hexane	81.10	4.7 × 10^–3^	53.33	0.08	63.04	1.7 × 10^–3^
hexatriene	93.08	6.9 × 10^–3^	53.17	0.12	64.16	1.9 × 10^–3^
styrene	78.39	7.3 × 10^–3^	44.07	0.12	55.44	1.5 × 10^–3^
isoprene	96.61	7.3 × 10^–3^	52.07	0.11	69.04	2.0 × 10^–3^

a The errors on MO energies and the Löwdin charges obtained from different models 1, 2, and 3 for minimal basis targets (Figure 1).  = MSE_H_ is the mean squared loss on the Hamiltonian optimized in model 1.  = MSE_ε_ is the mean squared loss on the MO energies optimized in model 2, and  = MSE_ε,*q*_ is the sum of mean squared losses on the MO energies and the Löwdin charges optimized in model 3.

### Large Basis Targets

**Figure 3:**
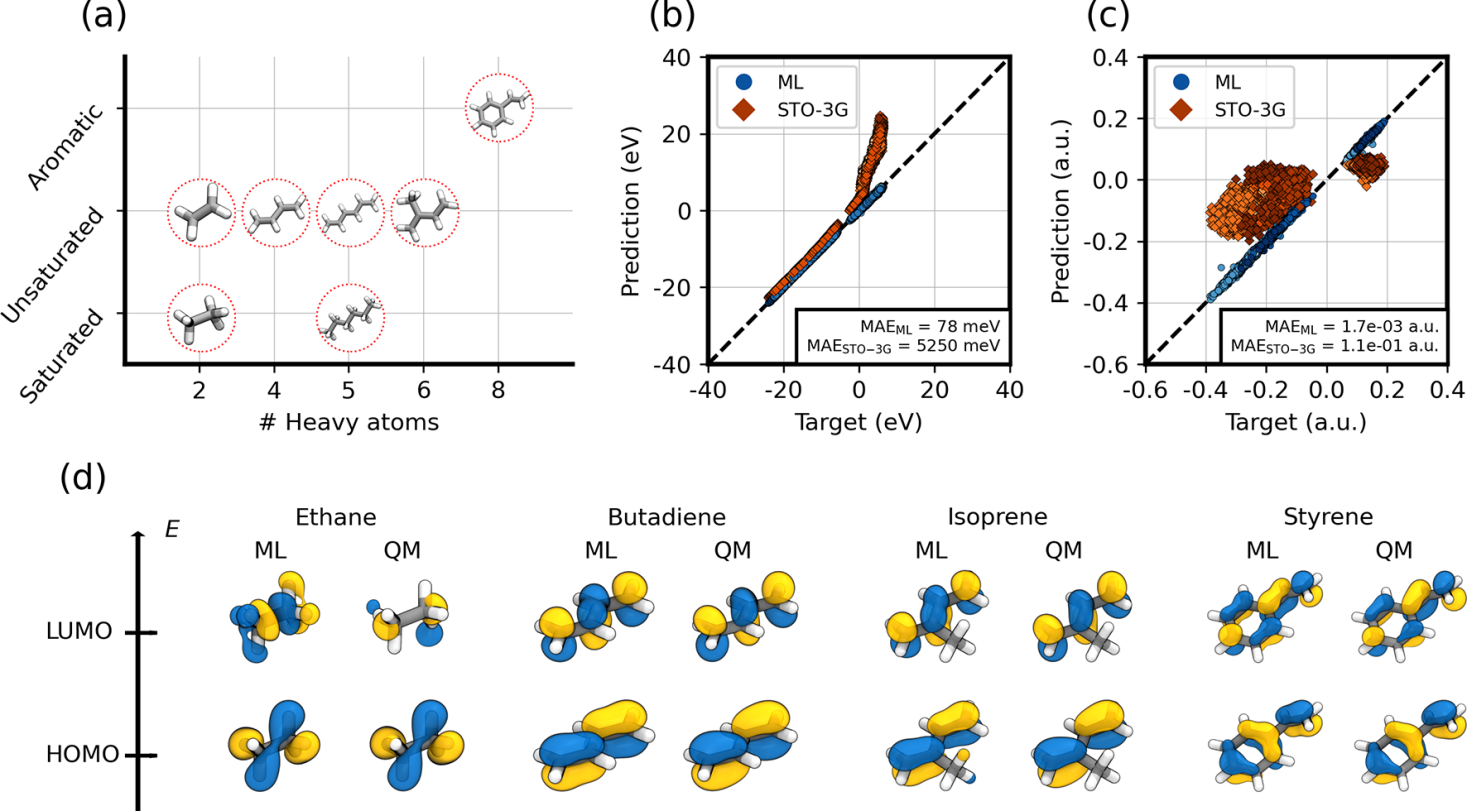
Performance of the ML model on large basis targets. (a) The hydrocarbons in the training data set: ethane, ethene, butadiene, hexane, hexatriene, isoprene, and styrene. The two panels on the right show the performance of the ML model on test geometries of the training molecules, for the MO energy (b) and the Löwdin charges (c). The target is always computed with B3LYP/def2-TZVP. ML predictions of the LBT model are indicated with blue points, the corresponding results of the STO-3G calculations are in orange. The mean absolute error (MAE) is reported for both. Different shades of blue and orange correspond to the different molecules (panel a). A detailed plot showing the prediction for each molecule separately is reported in Figure S6. (d) Comparison of HOMO and LUMO molecular orbitals between the ML prediction in a minimal pseudobasis and the target B3LYP/def2-TZVP.

The comparison between different architectures reveals the need to balance data- and physics-based considerations when optimizing the overall architecture of the ML model. Even though the accuracy of predictions for the indirect model (3) is remarkable, one has to keep in mind that a minimal basis QM description of the electronic structure is very far from converged: errors on the electronic eigenvalues, particularly for low-lying excited states, are often on the order of several electronvolts. As anticipated, an indirect training strategy can also be used to predict target quantities that are computed with larger basis sets, while keeping a model architecture consistent with a minimal basis. Specifically, we use as targets the valence-state eigenvalues ε_*n*_^LB^ and Löwdin charges *q*_*i*_^LB^ computed from a large triple-ζ basis (that contains 1s, 2s, 2p, and 3s orbitals for H and 1s, 2s, 2p, 3s, 3p, 3d, 4s, 4p, 4d, 4f, and 5s for C atoms). The performance of this model is shown in Figure 3 for both the MO energies and atomic charges (see Figure S6 for a detailed plot).

Compared to the minimal-basis predictions (see Table 1), the errors from the model targeting a large-basis Hamiltonian (LBT model) are considerably larger (i.e., up to a factor of 10 for ethane; see Figure S6 and Table S1). The larger inaccuracy of the LBT model is to be expected, as the target is far more complex than in the minimal-basis case. However, these errors are at least an order of magnitude smaller than the error of an explicit QM calculation in a minimal basis (see Figure 3b and c). Indeed, the average MAE on MO energies over the test conformations is 78 meV for the LBT model, to be compared with the 5250 meV for the explicit minimal-basis QM calculation (Figure 3b). This shows that the LBT model learns an effective pseudo-Hamiltonian that reproduces the desired electronic properties of an expensive QM calculation on a large basis to a high accuracy.

Thanks to the prediction of an electronic Hamiltonian, the LBT model retains some of the interpretability of a QM calculation. Indeed, it is possible to visualize the MO shapes to understand the quality of the prediction, as we show in Figure 3d for various molecules. We stress that even though the pseudo-Hamiltonian has the symmetries of a minimal basis **H**, it is not explicitly tied to a choice of atom-centered functions, and any basis with the correct symmetry can be used to visualize the MOs. In Figure 3d, we chose the STO-3G basis for simplicity. The LUMO orbital of ethane is the only one exhibiting a mismatch with the ML prediction. The difficulty in predicting this MO lies in its Rydberg character (Figure S7). Rydberg orbitals are known to be present for calculations of small molecules in the gas phase, especially when using atomic bases with diffuse orbitals. Their diffuse character appears to be particularly challenging for the LBT model. All of the other orbitals show that the symmetry and nodal structure of the LBT one-electron wave functions are learned correctly (see Figure 3d). For the remainder of this study, we will focus exclusively on this type of LBT hybrid models, which offers an excellent trade-off between accuracy and computational expense.

### Electronic Excitations from an ML Hamiltonian

**Figure 4:**
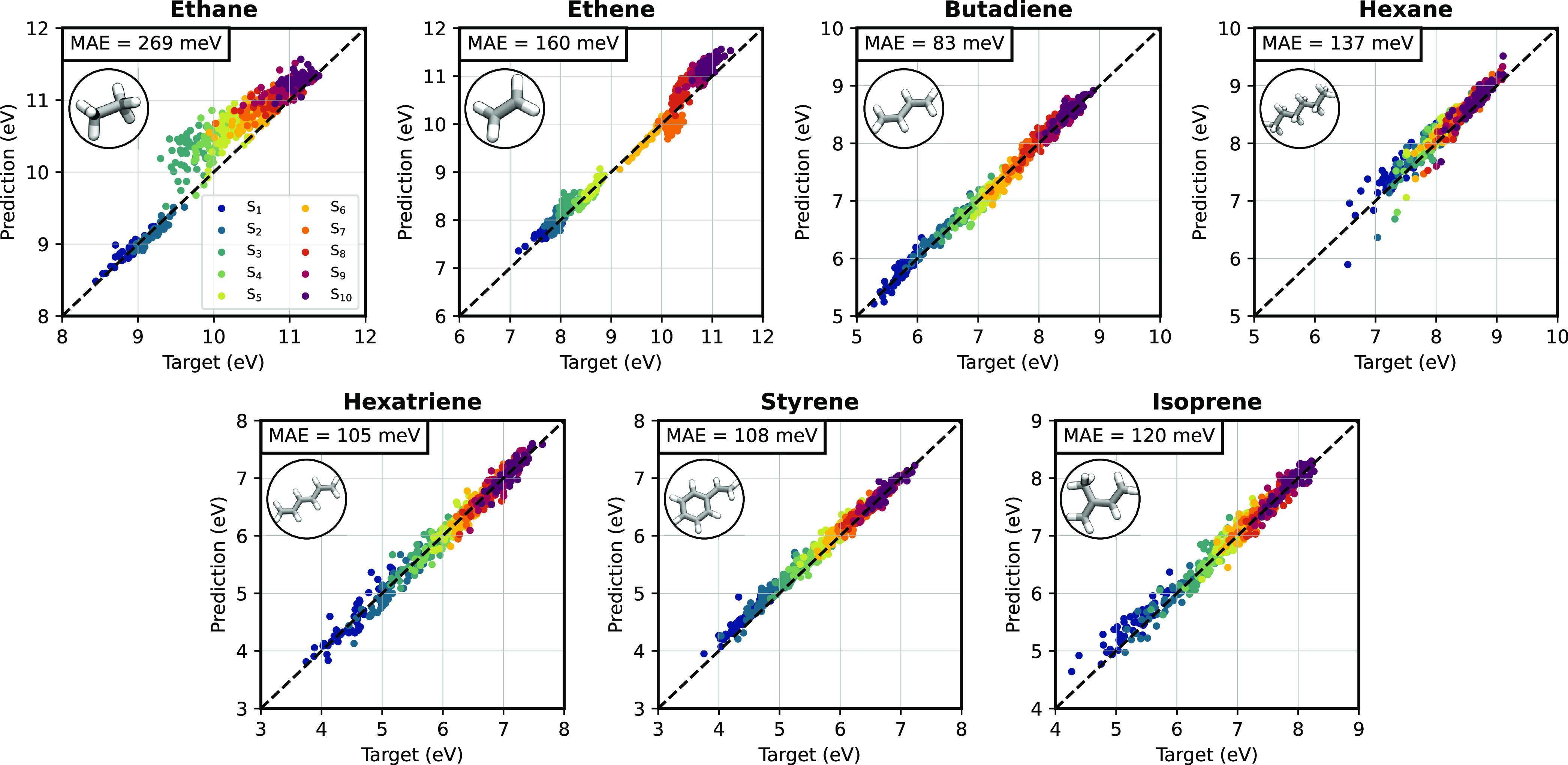
Prediction of the electronic excited states. Performance of the ML model on predicting the excitation energy for the first 10 singlet excited states. The target is computed with sTDA B3LYP/def2-TZVP. The prediction is computed by coupling the ML prediction with sTDA. The average MAE over all excited states is reported in the inset.

One of the advantages of explicit electronic-structure calculations is that, after having determined the self-consistent single-particle Hamiltonian, they allow the prediction of many molecular properties through simple and inexpensive postprocessing steps. Our benchmarks thus far only validate the accuracy for properties that are explicitly trained on. To check whether the predicted pseudo-Hamiltonian can also be manipulated to access other properties, we consider the problem of predicting excitation energies based on time-dependent DFT (TD-DFT), which is commonly used to compute excited states for large molecules. We use, in particular, an approximation of TD-DFT developed by Grimme^36^ called simplified Tamm–Dancoff Approximation (sTDA). Its appeal in our present case is that integrals in sTDA are approximated with Löwdin charges that are readily available from our ML prediction (see Methods). As shown in Figure 4, the excitation energies computed from the effective ML Hamiltonian are predicted with good accuracy, with a balanced error across the first 10 singlet excited states. The error increases as the molecule gets larger and more flexible, such as for hexane, but it always remains well below 200 meV except for ethane, for which Rydberg states cannot be properly captured by a minimal-basis Hamiltonian. As a point of comparison, excitation energies computed at the STO-3G level differ by at least 1 eV from those computed with a large basis set (see Table S2). We also note that the predictions for ethane and ethene, the smallest molecules in the training set, suffer from the presence of Rydberg orbitals that we have previously evidenced. What makes these results particularly remarkable is that the model does not explicitly use the sTDA excitations as targets, which indicates good generalization capabilities of the ML model and, at the same time, scope for further improvement of the accuracy by including these additional targets to the optimization step. Furthermore, as obtaining a balanced description of many excited states with ML is known to be challenging,^10^ our results show that learning a molecular Hamiltonian is a viable way for obtaining a consistent prediction of excited states.

**Figure 5:**
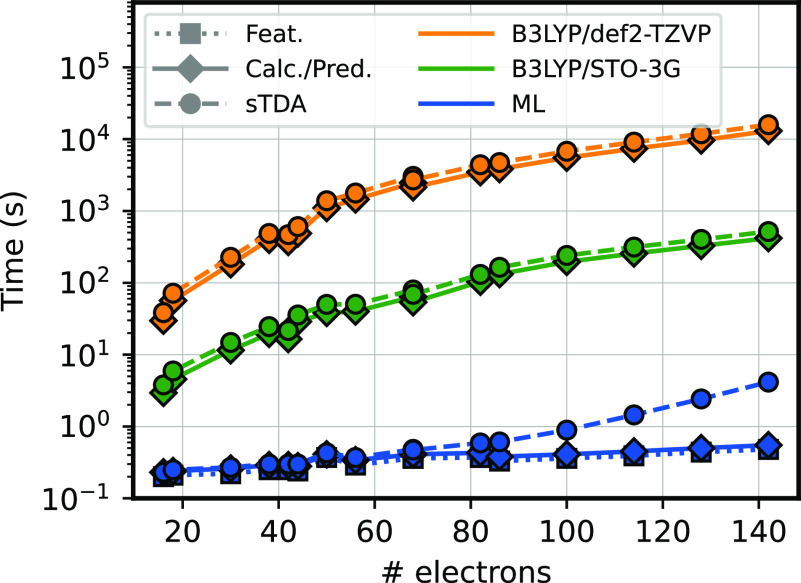
Timings of the ML model and QM calculations. Time to compute excited state energies for molecules with an increasing number of electrons. The B3LYP/def2-TZVP time (orange) and the B3LYP/STO-3G time (green) are computed as the sum of the DFT calculation and sTDA. The ML time (blue) is the sum of the featurization time, the prediction time, and the sTDA. Each time is computed on a single core of an Intel Xeon Gold 5120 Processor. B3LYP/def2-TZVP and B3LYP/STO-3G calculations are performed with PySCF. More details are in Table S3 in the SI.

Overall, our indirect learning strategy delivers predictions of electronic properties with an accuracy comparable to that of converged settings across many excited states at a cost that is much smaller than that of a minimal-basis DFT calculation and many orders of magnitude faster when compared to the large basis (see Figure 5). The speed gain is mainly due to the ML algorithm itself, which directly predicts the blocks of the self-consistent Hamiltonian. The minimal-basis formulation reduces the cost of the diagonalization step, although—for the larger molecules—evaluating the sTDA excitations becomes the rate-limiting step for predictions that start from the ML-based pseudo-Hamiltonian. The local nature of the model also means that the ML Hamiltonian is highly sparse, which guarantees linear-scaling predition of the pseudo-Hamiltonian in the large system-size limit and would be beneficial in combination with linear-scaling solvers of the eigenvalue problem.^39^ Even though the nature of the model ensures benign scaling and generalizability to more complex chemistry, we stress that the current implementation is not optimized for speed, being instead focused on making it easy to test new ideas: therefore, a more efficient calculation of symmetry-adapted features, as well as the use of more sophisticated model architectures, leaves much room to further improve the accuracy and computational requirements.

### Extrapolative Predictions

**Figure 6:**
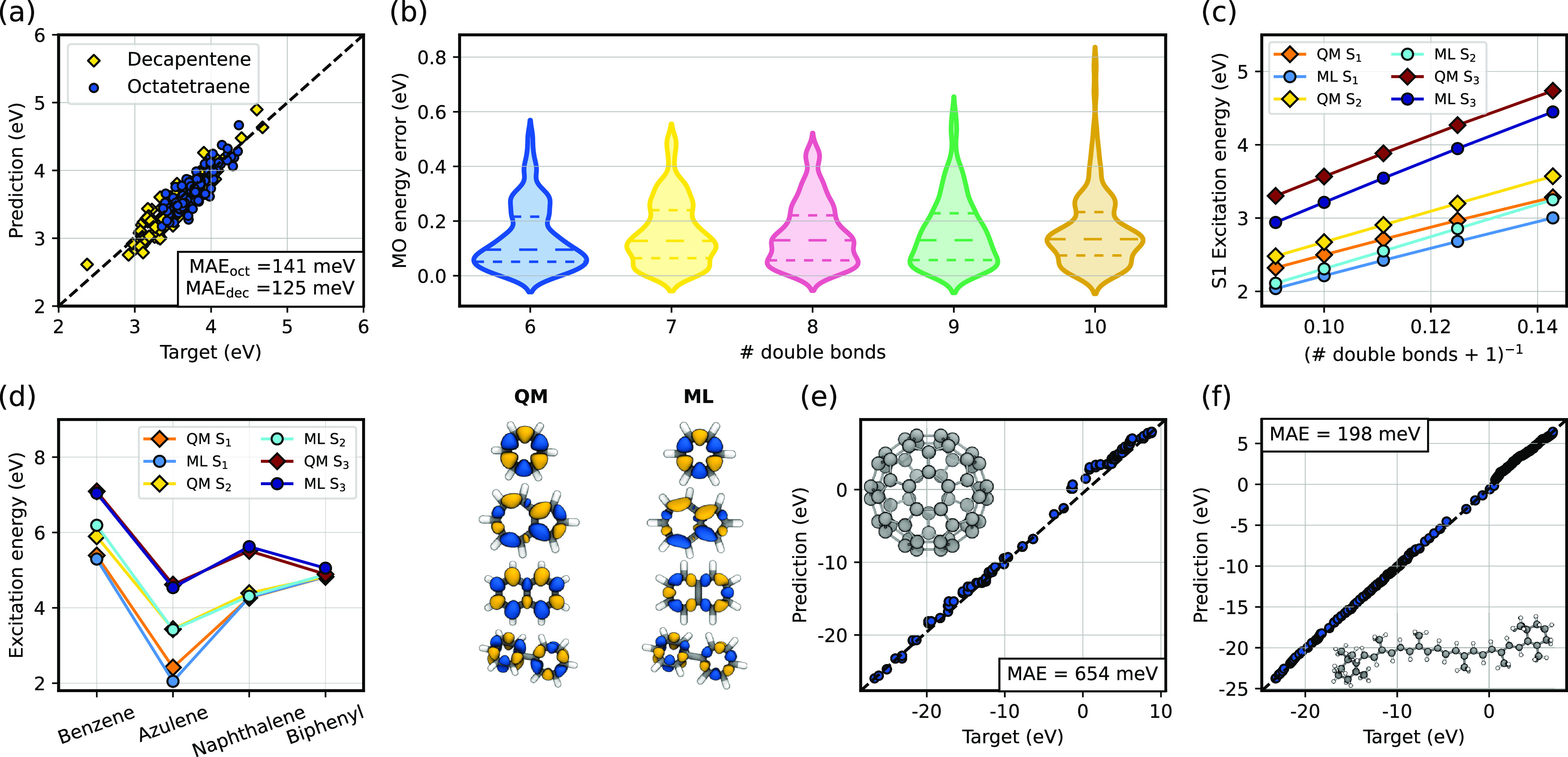
Generalization performance of the ML model. The QM target is B3LYP/def2-TZVP, coupled with sTDA to obtain the excitation energies. (a) ML prediction of the excitation energy of the first singlet excited state of octatetraene and decapentene. The MAE is reported for both molecules. The subscript “oct” refers to octatetraene and “dec” to decapentene. (b) Distribution of absolute errors for the MO energies of polyalkenes with a progressively longer conjugated chain. The dashed lines are the first, second, and third quartiles of the distribution. (c) ML prediction of the excitation energy of the first three singlet excited states for polyalkenes, from six up to 10 double bonds. (d) ML prediction of the excitation energy of the first three singlet excited states for some aromatic molecules. The transition density of the first excited state is visualized on the right. For the ML model, we have used the STO-3G basis to obtain the transition density cubes. (e) ML prediction of the MO energies of C_60_. (f) ML prediction of the MO energies of β-carotene.

The calculation of the excitation energies based on the ML-derived minimal-basis Hamiltonian demonstrates the ability of our framework to generalize to new *properties*, beyond those on which it has been trained. As we shall see, it also demonstrates excellent transferability to new *structures*, much larger and more complex than those included in the training set. As a first benchmark, we tested our ML model by predicting the excitation energies for polyalkenes of different lengths. For octatetraene and decapentene, we extracted 100 conformations from a REMD trajectory spanning a broad temperature range, following a protocol analogous to that used to generate the train set. Figure 6a shows the prediction of the first excited state for several conformations of octatetraene and decapentene. Errors are comparable to those observed in the validation set, indicating excellent transferability to bigger molecules. Indeed, Figure S10 shows that similar levels of accuracy can be achieved up to the tenth singlet excited state. The transferability of the ML model is further confirmed by the small errors obtained for the prediction of the MO energies (Figure 6b and Figure S8) of longer polyalkenes with up to 10 double bonds.

In this benchmark, errors are dominated by the presence of distorted configurations. The excitation energy for the optimized geometries (Figure 6c) provides clearer insights into specific physical effects without the noise introduced by thermal distortion. The model perfectly captures the dependence of the first three excited states on the increase in conjugation length, but there is a redshift of approximately 300 meV relative to the target values. This redshift illustrates a limitation of the ML framework, which uses short-ranged features to parametrize the pseudo-Hamiltonian, and all matrix elements involving atoms beyond the cutoff are predicted to be zero (Figure S9). This effect results in a systematic underestimation of the HOMO–LUMO gap (Figures S9 and S11), which translates into an underestimation of the excitation energy. Increasing the cutoff of the ML features would be an obvious strategy to address this problem, which however also leads to a model that is less accurate and transferable, as it would require training on larger molecules to thoroughly sample longer-range interactions (see Figure S2). An alternative, very effective solution is to use an explicit minimal-basis calculation as a baseline, using the ML model to learn the large-basis targets by correcting the STO-3G Hamiltonian. As shown in the SI, this baselined model yields much lower errors and completely eliminates the redshift. This is relevant in light of several recent efforts that use low-cost electronic-structure properties as molecular descriptors^40,41^ and representative of the kind of trade-offs that are necessary when designing hybrid modeling schemes. It entails, however, a substantial increase in computational cost, and we will restrict our investigation to a model that does not rely on a baseline.

We also consider the case of four representative aromatic molecules, none of which is included in the training set (Figure 6d). Even though styrene is the only aromatic molecule used during training, the prediction of the first three excited states for the test aromatic molecules matches very well the reference values. Azulene, which contains a pentagonal motif that is completely missing in the training data, shows the largest error in the first excited state. Figure 6 also shows the transition density associated with the first excited state for both the ML prediction and the target. The ML-based transition density matches well the qualitative nature of the reference, indicating that the model predicts the correct excitation despite small quantitative differences in the shape due to the use of a minimal basis set. Naphthalene is the only exception: for this molecule, the L_*a*_ and L_*b*_ states are particularly challenging for computational methods:^42^ for B3LYP/def2-TZVP, in particular, they are very close in energy (see Figure 6d). As a result, the small errors in the ML model lead to an exchange in their ordering (Figure S12).

These results, together with the ability of the model to capture the correct trend in the excitation energy of polyenes, demonstrate that using a hybrid model based on the evaluation of an effective Hamiltonian as an intermediate step allows us to capture global effects such as conjugation and aromaticity, despite being modeled from local features. This is in stark contrast to models that target the excitation energy directly, which would not be capable of capturing changes for molecules larger than those included in the training set (see, e.g., ref (44) for an example of this effect in the case of the molecular polarizability). An analysis of the dependence of the excitation energies of biphenyl and butadiene as a function of molecular distortions confirms that a sTDA calculation based on the ML Hamiltonian correctly captures the qualitative behavior of excited states and is more accurate relative to the converged DFT calculations than commonly used semiempirical methods, even when applied in an extrapolative regime (Figure S14).

As a final example, we test our model to predict the Hamiltonian of very large molecules, namely, C_60_ fullerene and β-carotene (Figure 6e and f). Despite its size and complexity, MO energies of β-carotene are predicted with an MAE of less than 200 meV. This error is largely due to the underestimation of the HOMO–LUMO gap, similar to what we observed in much simpler, linear polyalkenes (Figure 6c). The peculiar structural features of C_60_, with the presence of pentagonal rings, a complete lack of hydrogen atoms, and high curvature, make it a more extreme outlier relative to the training set. Nevertheless, the LBT model achieves an MAE of 654 meV on the MO energies, which is much smaller than the error of a minimal-basis calculation (MAE in excess of 4.7 eV). For β-carotene the atomic Löwdin charges are predicted with an accuracy comparable to that observed for small molecules, while for C_60_ they are exactly zero due to symmetry (Figure S13), which is captured by the ML model because of its equivariant structure.

### Vibronic Spectra

As a final application, we demonstrate how our hybrid model can be further combined with advanced simulation techniques to obtain accurate, yet inexpensive, predictions of subtle quantum-mechanical effects. We estimate the vibronic spectrum of anthracene via second-order cumulant expansion theory (see Methods). Within this framework, the vibronic structure of the excitation is encoded by the spectral density. Among the different approaches to compute this quantity,^45,46^ we rely on the calculation of the autocorrelation function of the excitation energy along an MD trajectory. This dynamical method is typically very accurate,^47^ and transparently incorporates vibrational information. This is an application where a fast surrogate ML model for excited states can make the results of prohibitively demanding quantum-chemical calculations accessible: a single sTDA calculation for anthracene at the B3LYP/def2-TZVP level requires approximately 8 CPU minutes, versus half a second for the hybrid model, a speed-up of 3 orders of magnitude.

**Figure 7:**
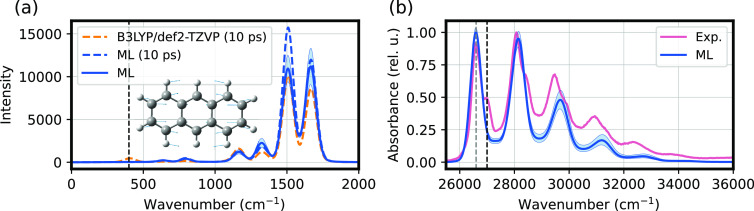
Vibronic spectrum of anthracene. (a) Spectral density predicted with the ML model (solid blue line) and corresponding 95% confidence interval. The spectral density is an average over several windows of 100 ps of MD trajectory. The spectral density for the target sTDA B3LYP/def2-TZVP (orange dashed line) is reported for a single window of 10 ps, alongside the corresponding ML prediction (blue dashed line). (b) Absorption spectrum predicted with the ML model (blue) and compared with the experimental spectrum^43^ (orange). The ML spectrum is the average over 100 ps of MD for a gas-phase anthracene molecule. The blue shaded region denotes the 95% confidence interval around the mean. The vertical dashed black line in panel a denotes a peak around 400 cm^–1^ that is not captured by ML. The black dashed line in panel b, shifted by 400 cm^–1^ from the gray line, denotes the corresponding missing vibronic shoulder. The normal mode at around 400 cm^–1^ is shown in the anthracene sketch in panel a.

The spectral density and vibronic spectrum of anthracene are shown in Figure 7a and b, respectively. The low cost of ML calculations allows us to average the spectral density over several 10 ps windows. The corresponding averaged spectral density for sTDA B3LYP/def2-TZVP is much more demanding, and we show its value computed for a single window. The target spectral density is mostly within the confidence interval reported for the ML averaged spectral density (blue interval; Figure 7a). A comparison with the ML spectral density computed in the same window shows that all of the peaks are slightly overestimated by the ML, with the exception of a peak at around 400 cm^–1^ that is absent in the ML prediction. This peak is responsible for the vibronic shoulder visible in the experimental absorption spectrum, which is also missing in the one predicted from ML simulations (Figure 7b and Figure S16). Inspection of this normal mode shows that it is a global “breathing”-like motion of the entire molecule (see inset in Figure 7a) arising from small alterations of the interatomic distances in anthracene and thus hard to characterize with short-ranged features. Besides this minor discrepancy, the absorption spectrum is in good agreement with the experimental one. Indeed, the ML spectrum shows a competitive performance with ZINDO (Figure S15), a similarly fast semiempirical method specifically built to target singlet excited states of organic molecules and sometimes used to compute spectral densities in complex biomolecules.^48,49^

## Discussion

At the most fundamental level, the difference between physically motivated and data-driven modeling approaches is that of primarily deductive and naively inductive paradigms of scientific knowledge. The former strives for universality and to reduce the complexity of empirical observations to a minimal set of fundamental laws, while the latter infers patterns from data and is usually more limited in generalization power but can be more precise, or computationally efficient, in capturing structure–property relations. The approach we discuss here to describe electronic excitations, combining machine-learning of an effective minimal-basis, single-particle Hamiltonian with training on a large basis set and the further application to evaluate physics-based approximations of molecular excitations, demonstrates the advantages of a middle-ground, hybrid solution.

Despite the relatively small training set composed of MD snapshots of seven small hydrocarbon molecules and the simplistic choice of ML architecture, our model shows excellent transferability, both in terms of the evaluation of derived electronic properties and in terms of making predictions for larger, more complex compounds. We probe the former aspect by computing excitation energies in the sTDA approximation or the vibronic spectrum based on molecular dynamics trajectories and the latter by making predictions on molecules that are larger and more complex than those included in the training. In both cases, we demonstrate excellent transferability, with similar errors observed in the interpolative and extrapolative regime—except for cases, such as C_60_, which entail dramatically different chemical motifs. In every case we consider, we obtain an accuracy that is much better than that afforded by an explicit minimal-basis quantum calculation at a considerably reduced cost. We capture qualitative physical effects, such as the dependence of molecular excitations on the extent of conjugation or on internal molecular rotations. We can also easily interpret the quantitative errors, which we trace back to the aggressive local truncation of descriptors or to the difficulty of treating delocalized Rydberg states using a minimal basis.

We make a few observations that could guide the development of similar, hybrid models. (1) Reproducing the mathematical structure of the quantum mechanical approximations is more effective than explicitly targeting the value of approximate electronic-structure quantities. (2) Using indirect properties as targets, such as single-particle eigenvalues, makes it possible to “promote” the model accuracy to a higher level of theory, e.g., using a larger basis set, at no additional cost. (3) Indirect learning should be sufficiently constrained to avoid overfitting and unphysical predictions. (4) The use of well-principled descriptors, that incorporate the symmetries of the problem, is beneficial in reproducing the qualitative behavior of the excitations. (5) Locality plays a fundamental role in facilitating transferability, but there is a trade-off with the asymptotic accuracy that the model can achieve. One of the main challenges is that, despite the shared features,^24,50^ the design space of ML models for chemistry is very large.^51^

Incorporating physical approximations into an ML model can make the architecture easier to interpret but also introduces more degrees of freedom that need to be explored. We suggest that restricting the investigation to simple, easy-to-interpret ML models, translating some of the insights that have become well-established in the construction of data-driven interatomic potentials to electronic-structure targets, and emphasizing generalization power over in-sample benchmark accuracy, which is the main advantage of physics-based, deductive modeling, are the next logical steps in advancing the integration between machine learning and quantum chemistry.

## Methods

### Model Architecture

Our ML model aims to reproduce the single-particle states (MOs) from a mean-field quantum chemistry calculation such as Hartree–Fock or Kohn–Sham DFT. These MOs are expanded on a basis set of atomic orbitals (AOs), and their coefficients are obtained from the solution of the self-consistent-field (SCF) generalized eigenvalue equation:
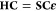
1where **H** is the Fock (or Kohn–Sham) matrix, **S** is the overlap matrix of the AO basis, and **ε** is a diagonal matrix containing the MO energies. As **H** depends on the orbitals themselves, the iterative solution of this equation is numerically expensive, especially for large basis sets.

We learn an effective Hamiltonian **H̃** that substitutes for **H** in the eigenvalue equation and directly yields the MOs, bypassing the SCF solution. To simplify and constrain the problem, we require that **H̃** be defined on a minimal and orthonormal AO basis, akin to standard semiempirical methods. In addition, the minimal basis over which we learn **H̃** is only implicitly defined, a feature that improves the model flexibility. We train our model so that the solutions
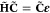
2generate a selected subset of MOs and MO energies as the original SCF equations. The obtained MO coefficients and energies can be used to predict additional quantities, such as electronic excitation energies, within a physics-based model.

For model 1, which directly targets the entries of an orthogonalized Hamiltonian, we use Löwdin-symmetrized Fock **H**_LSF_ = **S**^–1/2^**HS**^–1/2^ computed with B3LYP/STO-3G as our target. When we target an SCF Fock **H** in a larger basis, which yields more accurate results, we cannot link its entries to our prediction **H̃** in an implicit minimal basis. Instead, we ensure that our model generates the desired subset of MOs, with energies that are as close as possible to the quantum chemical calculations. To do so, we first train our model indirectly on B3LYP/STO-3G targets, minimizing a loss of MO energies (model 2) and of MO energies and Löwdin charges (model 3). The Löwdin charge *q*_*A*_ on atom *A* is computed as
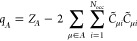
3where *Z*_*A*_ is the atomic number of *A*, μ indexes an atomic orbital, *N*_occ_ is the number of occupied MOs, and the MO coefficients **C̃** are obtained from eq 2. The final LBT ML model is trained on MO energies and Löwdin charges, as for model 3, but on targets computed with B3LYP/def2-TZVP.

### Data Set Generation

We use a data set of 1000 different geometries of ethane, ethene, butadiene, and octatetraene that was originally presented in ref (8). We extend it with configurations of hexane, hexatriene, isoprene, styrene, and decapentene following a similar protocol to that in the reference. In summary, we carry out a replica exchange molecular dynamics (REMD) simulation with a time step of 0.5 fs, for a total of 150 ps of sampling per replica and attempting exchanges every 2 fs. Molecular dynamics trajectories for each replica were integrated in the constant-temperature ensemble by using a generalized Langevin equation (GLE) thermostat. Forces were computed at the DFTB3-UFF/3OB level of theory. The simulation was run with i-PI^52^ in combination with DFTB+ .^53^ For each molecule, 1000 structures were selected from all trajectories, using farthest point sampling (FPS)^54^ on SOAP^55^ descriptors averaged over the structures. The SOAP descriptor was computed with rascaline,^56^ using a cutoff of 4.5 Å, *n*_max_ = 6, *l*_max_ = 4, and a Gaussian width of 0.2 Å. FPS was performed with scikit-matter.^57^ For these data sets, we then performed DFT calculations using both the STO-3G and def2-TZVP basis sets and Gaussian-like B3LYP functional (b3lypg) level of theory using PySCF,^58^ to obtain the Fock and overlap matrices and other required electronic structure properties. All calculations were performed on a spherical atomic basis, and molecular symmetry was not taken into account even when present, as for some optimized geometries.

### Symmetry-Adapted Hamiltonian Regression

The single-particle Hamiltonian is expressed in terms of AO basis functions ϕ_*a*_(**x** – **r**_*i*_), where *a* = *ñl̃m̃* denotes the orbital symmetry and **r**_*i*_, the atom on which the orbital is centered. We use the shorthand ⟨*ia*|*Ĥ*|*jb*⟩ = *H*_*ia*,*jb*_(*A*) to denote the Hamiltonian matrix element between an orbital ϕ_*a*_ centered on atom *i* and ϕ_*b*_ centered on atom *j* of a structure *A*. Due to the presence of two atomic indices, these elements must be equivariant to permutations of the orbital labels associated with each atomic center. The rotations of each block of the matrix (involving all the corresponding *m* and *m*′ indices) can be decomposed into rotations of irreducible representations (irreps) of *O*(3) (the transformation from the uncoupled |*l̃m̃*⟩|*l̃*′*m̃*′⟩ basis to the *coupled* angular basis (irrep) |λμ⟩ is effected through Clebsch-Gordan coefficients). We model each irreducible block separately, so that our targets have the form *H*_*ij*_^*p*τλμ^ where (λμ) is the SO(3) symmetry index, τ captures additional symmetries (e.g., inversion parity) associated with this block, and *p* enumerates the other variables, i.e., the angular (*l̃*,*l̃*′) and radial (*ñ*,*ñ*′) basis, as well as the chemical nature of the atoms. Given this symmetry-based decomposition of **H**, we can employ ML models of arbitrary complexity as long as they are equivariant to the same permutation and SO(3) symmetries. Here, we use linear models with descriptors ξ^τλμ^(*A*_*ij*_) with the same symmetries of the target:
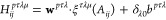
4where **w**^*p*τλ^ are invariant weights and δ_λ0_*b*^*p*τλ^ is the intercept for the scalar (λ = 0) blocks, which in our model we take to be zero. We stress here that this framework, while described for linear models, is equally compatible with any equivariant deep-learning scheme.

### Symmetry-Adapted Features

Our descriptors ξ^τλμ^(*A*_*ij*_) are the two-centered features described in ref (24) obtained as generalizations of the atom-centered density correlation descriptors^55,59,60^ to simultaneously represent multiple atomic centers and their connectivity. Briefly, these features rely on the *pair* density coefficients *c*_*nlm*_(*A*_*ij*_) as the core ingredients that essentially specify the position of atom *j* relative to atom *i*.
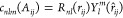
5In this form, the spatial description has been discretized on a more tractable basis *R*_*nl*_ (GTO-style radial functions) and spherical harmonics *Y*_*l*_^*m*^(*r̂*) for the radial and angular degrees of freedom, respectively, as is commonly done in quantum chemistry codes.

Summing over one of the indices (*j*) for all pairs within a set cutoff distance leads to a neighbor density *c*_*nlm*_(*A*_*i*_) = *∑*_*j*_*c*_*nlm*_(*A*_*ij*_), which describes the local correlations of a single center *i* and its neighbors. Combinations (through tensor products) of the neighbor density express higher-order correlations with multiple neighbors. On the other hand, the combination of the neighbor density with *c*_*nlm*_(*A*_*ij*_) yields a richer description of the specific pair between two atoms (*ij*). In particular, the simplest such combination *c*_*n*′*l*′*m*′_(*A*_*i*_)*c*_*nlm*_(*A*_*ij*_) describes the correlations of three atoms–two centers *i* and *j* and the neighbors of *i*. The cutoff distance enforces locality of the descriptor and usually is an optimizable hyperparameter; however, a cutoff smaller than the interatomic separation (between *i* and *j*) means that there will be no features corresponding to the pair and hence a zero prediction for all the associated Hamiltonian blocks (see e.g. Figure S3). The choice of spherical harmonics as the angular basis and implementation of the combinations (tensor products) through a Clebsch–Gordan coupling (similar to the one described for the Hamiltonian blocks) ensures rotational equivariance of the features, and symmetry to the permutation of the atom labels can be similarly enforced by averaging over the permutation group. We direct the interested reader to ref (24) for more details about the symmetrization of the features.

### sTDA

Excitation energies were computed using Grimme’s simplified Tamm–Dancoff Approach (sTDA),^36,37^ solving the TDA equations **AX** = Ω**X**, where **X** indicates the configuration interaction singles (CIS) amplitudes that describe the excitations. sTDA uses an approximated form for **A**, in which exchange-correlation terms are neglected and integrals are simplified:
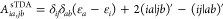
6where ε_*i*_ is the MO energy for the *i*th orbital, the *i* and *j* indices refer to occupied orbitals, and *a* and *b* refer to virtual orbitals. Integrals are evaluated using a monopole approximation:
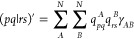
7where *q*_*pq*_^*A*^ is the Löwdin transition charge between MOs *p* and *q* for atom *A*, the sums run over all the atoms of the molecule, and γ_*AB*_ is a Matanaga–Nishimoto–Ohno–Klopman term.^36^ The Löwdin transition charges are computed from the predicted MO coefficients **C̃** as
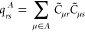
8where the sum runs over atomic orbitals μ centered on atom A. Additional approximations are present in sTDA to speed up the calculation. The CI space is truncated by using a user-defined threshold, followed by an additional selection of the most important electronic configurations to be included. For details, we refer to the original publication.^36^

All of the ingredients needed for sTDA (namely, the MO energies and the transition Löwdin charges) are available from the ML model, which makes the coupling of the ML model to sTDA straightforward. All our calculations are performed with an in-house implementation of sTDA in a spherical basis^61^ in PyTorch.^62^ For both polyalkenes (from five to 10 double bonds), aromatic molecules (benzene, azulene, biphenyl, naphtalene), β-carotene, and C_60_, the geometry was optimized with DFT at the B3LYP/6-31G(d) level of theory using Gaussian.^63^

### Vibronic Spectra

The anthracene trajectory was generated with DFTB3/3OB dynamics in the NVT ensemble with the Langevin thermostat and a coupling constant of 1 ps^–1^. The temperature was set to 300 K. We used a time step of 0.5 fs, for a total simulation time of 100 ps. Coordinates were saved every 1 fs. The simulation was run with AMBER.^64^ Vibronic spectra were computed using the second-order cumulant expansion formalism.^65,66^ Starting from a correlated trajectory, the excitation energy *U*(*t*) is computed for each frame and used to evaluate its autocorrelation function *c*_*UU*_(*t*) = ⟨*U*(*t*)*U*(0)⟩. The autocorrelation is used to calculate the spectral density function *J*(ω) encoding the vibronic coupling:
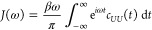
9The autocorrelation was damped in order to fall smoothly to zero in a time window of 10 ps. The vibronic homogeneous line shape is obtained from the spectral density through the line shape function *g*(*t*):
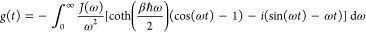
10from which the homogeneous absorption line shape is computed as
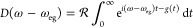
11Finally, the absorption spectrum is obtained after incorporating into the homogeneous spectrum static disorder, modeled by random sampling from a Gaussian distribution with a full width at half-maximum (fwhm) of 400 cm^–1^. The absorption spectrum was computed with SPECDEN.^67^

## Data Availability

All the software used in this study is freely available in the publicly accessible repositories halex^68^ and stda_torch.^61^ Reference data sets including molecular configurations and electronic-structure properties are available on the Materials Cloud archive.

## Supporting Information Available

Details on the training of ML models; prediction accuracy when using features with a larger cutoff; details and evaluation of the ΔML model; performance of the LBT model on predicting properties for test conformations of molecules present in the training; visualization of the Rydberg LUMO orbital for ethane; MO energy spectrum for polyalkenes; prediction of the first 10 singlet excited states for octatetraene and decapentene; analysis of the effect of the cutoff on the HOMO–LUMO gap of polyalkenes; transition densities for the first and second excited states of naphthalene; prediction of Löwdin charges for C_60_ and β-carotene; prediction of the excitation energy along a rigid scan on butadiene and biphenyl and comparison with ZINDO; spectral density and vibronic spectrum for anthracene computed with ZINDO; comparison of the predicted and target vibronic spectra for anthracene; comparison of the performance of the ML model targeting a minimal-basis and a large-basis Hamiltonian; errors on the first and second excited states for the seven hydrocarbons used to train the LBT model; timings for featurizing a molecule, predicting the Hamiltonian, and running sTDA (PDF)
Transparent Peer Review report available (PDF)
