# Supplementary material for: Electronic Excited States from Physically Constrained Machine Learning
Source: ACS Cent Sci. 2024 Feb 29;10(3):637–48. doi: 10.1021/acscentsci.3c01480 (PMC10979507; doi:10.1021/acscentsci.3c01480)
Supplement: Supplementary file 1 — oc3c01480_si_001.pdf [file oc3c01480_si_001.pdf]

Supporting Information: Electronic excited states from physically-constrained machine learning

Edoardo Cignoni,[†,§] Divya Suman,[‡,§] Jigyasa Nigam,[‡] Lorenzo Cupellini,[†] Benedetta Mennucci,[†] and Michele Ceriotti[*,‡,¶]

†*Dipartimento di Chimica e Chimica Industriale, Università di Pisa, Pisa, Italy*

‡*Laboratory of Computational Science and Modeling, Institut des Matériaux, École Polytechnique Fédérale de Lausanne, 1015 Lausanne, Switzerland*

¶*Division of Chemistry and Chemical Engineering, California Institute of Technology, Pasadena, CA, USA*

§*These authors contributed equally to this work*

E-mail: michele.ceriotti@epfl.ch

S1 Model Details and Training

We provide here the details of how we have trained the ML model presented in the main text. We have used as features the atom-centered two neighbor correlations obtained as the product of $c_q(A_i) = c_{nlm}(A_i)c_{n'l'm'}(A_i)$ with itself to describe the local environment around atom i and the corresponding three-body correlation descriptor $c_q(A_{ij}) = c_{nlm}(A_i)c_{n'l'm'}(A_{ij})$ to describe the pair of atoms (ij). As explained in the Materials and Methods and in Ref.,[S1] these features have been built iteratively by first expanding the atomic or pair density in a basis of radial functions and spherical harmonics, and then using combinations through

Clebsch-Gordan iterations to build the desired equivariant features (NICE framework).[S2,S3] The spherical expansion has been computed with `librascal`.[S4] In particular, the expansion was computed with six radial basis functions and using up to $l = 4$ real spherical harmonics. We have used a cutoff of 3.5 Å, using the default shifted cosine smoothing function with a smoothness width of 0.5 Å. The density was built using Gaussians with a width of 0.2 Å. It is important to note here that we have used quite a small cutoff of 3.5 Å while computing our descriptors, despite having much larger molecules in our training dataset.

The features are further post-processed, prior to their use in the ML model, with Principal Component Analysis (PCA). In particular, we have retained, for each symmetry block of the features, up to 200 principal components. This helps reduce some degrees of redundancy in the calculated features, and to lower the memory requirements of computing these descriptors. In order to preserve equivariance, a separate PCA is fitted, for each symmetry block in the features, without centering them. Indeed, feature centering is akin to fitting the intercept in a linear model, which breaks the equivariance property for all $\lambda > 0$.

Before starting the training with backpropagation on MO energies and Löwdin charges, an analytical ridge regression model is fitted to the elements of the STO-3G Fock matrix. This provides a sensible initial guess for the ML Fock matrix, greatly facilitating the training.

Starting from this fitted ridge guess, the ML parameters are further fitted by backpropagation via gradient descent. All our linear models are fitted without the intercept, even for the $\lambda = 0$ blocks of the Fock Hamiltonian. We have used a composite loss formed by a mean squared error (MSE) on MO energies and Löwdin atomic charges:

$$\mathcal{L}^{\varepsilon,\mathrm{q}} = \frac{\omega_\varepsilon}{N} \sum_{n=1}^{N} \frac{1}{O_n} \sum_{o=1}^{O_n} (\varepsilon_{no} - \tilde{\varepsilon}_{no})^2 + \frac{\omega_q}{N} \sum_{n=1}^{N} \frac{1}{M_n} \sum_{n=1}^{M_n} (q_{nm} - \tilde{q}_{nm})^2 + \omega_r \sum_{s=1}^{S} \frac{\|\boldsymbol{\omega_s}\|^2}{N_s} \tag{S1}$$

where N is the number of training points, O_n is the number of occupied MO orbitals in the n-th molecule, ε_{no} is the target MO energy, M_n is the number of atoms in the n-th

molecule, and q_{nm} is the target atomic charge. The tilde in $\tilde{\varepsilon}_{no}$ and $\tilde{q}_{nm}$ denotes the model prediction. ω_ε and ω_q are two hyperparameters weighting the relative importance of the two MSE losses. They have been determined so that, at convergence, the two losses have a similar magnitude and are of the order of unity. In this way, both terms have equal weight in the total loss. In particular, we have used $\omega_\varepsilon = 1.5 \times 10^6$ and $\omega_q = 1 \times 10^6$. The last term is an L2 regularization term. S denotes the number of symmetry blocks in the target, N_s is the number of samples in the s-th symmetry block, and $\boldsymbol{\omega}_s$ is the vector of linear weights for the s-th symmetry block. In this work we have used $\omega_r = 1 \times 10^{-14}$.

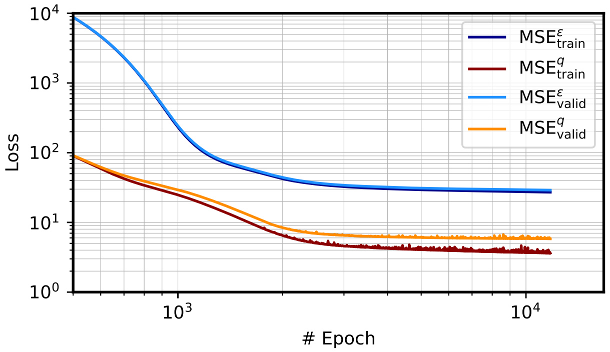

Figure S1: Loss versus Epoch curves for training and validation losses. The MSE on MO energies ε (first term in Eq. (S1)) and the MSE on Löwdin charges (second term in Eq. (S1)) are shown separately.

The model was trained on a training dataset composed of ethane, ethene, butadiene, hexane, hexatriene, isoprene, and styrene. For each molecule, a total of 250 conformations sampled from a REMD simulation (see Materials and Methods) have been used for training, for a total of 1750 training points. A validation dataset comprising 50 conformations per molecule, for a total of 350 conformations, has been used together with the training dataset to detect possible overfitting. The training was performed in batches of 100 conformations, using the Adam optimizer implemented in PyTorch with default parameters and a learning rate of 1. The model was trained until the loss converged to a plateau, where the training was interrupted. Training with these settings required about 9.5 s for one epoch, and between one and two days for the model to converge. The loss in the train and validation set is shown

in Figure S1.

S2 Training with a Larger Cutoff

We train our models with features computed for atoms within a cutoff distance of 3.5 Å. This translates into the impossibility of predicting matrix elements for atoms that are outside the cutoff. While this is surely a limitation when the interaction between distant atoms becomes significant, it is also beneficial from a model regularization perspective. In fact, a small cutoff to compute the features, reduces their complexity, and allows for better transferability and robustness of our models.

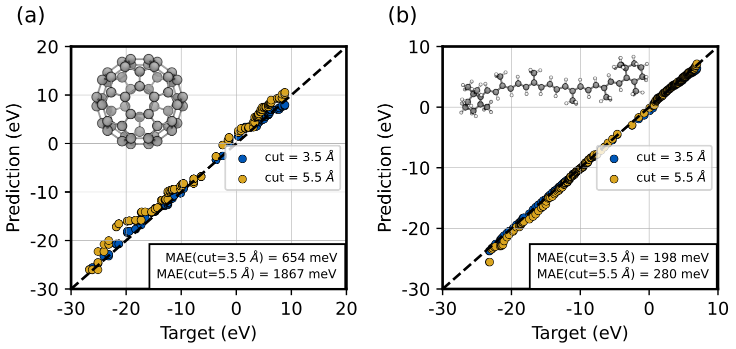

Figure S2: Effect of using a cutoff to compute features on the model performance. The mean absolute errors (MAE) when features are computed with a large cutoff compared to a smaller cutoff, for (a) C60 and (b) β-carotene. MAE is reported by including the valence MOs only. The MAE when core MOs are included is considerably larger.

This can be confirmed by training a model with features computed with a larger cutoff and comparing its performance on extrapolated molecules with that of a model trained using features with smaller cutoff.

Here, we target the B3LYP/def2-TZVP Hamiltonian, using features with a cutoff of 3.5 Å (the model shown in the main text) and with a larger cutoff of 5.5 Å. This latter cutoff is only moderately larger than the first, and many elements of the resulting Hamiltonian are still strictly zero. Nonetheless, the model exhibits clear signs of overfitting when it comes to extrapolating to completely out-of-sample molecules as we can see in Figure S2. The

errors for C60 and β-carotene increase significantly with larger cutoff. Thus, the use of a larger cutoff results in more powerful models that, if not trained extensively with a very large dataset, can lead to overfitting and loss of overall generalizability of the model.

S3 A Δ-ML model for the Electronic Hamiltonian

In the main text, as well as in the preceding section, we discussed the limitations associated with using a small cutoff for features while training a model and also highlighted how using a larger cutoff instead is detrimental to the model's generalization capability.

It is also known that the use of local features poses certain problems when long-range interactions become important.[S5–S7] While this limitation can be lifted in future refinements of the model through the use of long-range features,[S8] we show here an alternative approach to mitigate the limitations arising from the use of a small cutoff by ensuring the inclusion of long-range interactions through the implementation of a Δ-ML model.

We use the B3LYP/STO-3G QM calculation as our baseline model from which we obtain a zeroth-order Fock matrix. This baseline is then corrected with a Δ Fock matrix learned with ML in the very same way as the model discussed in the main text. We are therefore able to include long-range interactions within the Fock matrix at a lower cost since they are computed with a low-level QM calculation. A similar strategy has been reported in Ref.,[S9] although here it is used to learn an effective Fock with a minimal basis size and symmetries, targeting the properties of a Fock in a larger basis.

We denote the baseline Fock matrix obtained with e.g. STO-3G as $\mathbf{H}^{\text{base}}_{\text{QM}}$, and the Δ Fock matrix learned with ML as $\mathbf{H}^{\Delta}_{\text{ML}}$, the Fock matrix $\mathbf{H}$ predicted by the model is given as :

$$\mathbf{H} = \mathbf{H}^{\text{base}}_{\text{QM}} + \mathbf{H}^{\Delta}_{\text{ML}} \tag{S2}$$

This matrix is diagonalized to obtain MO energies and Löwdin charges, and the $\mathbf{H}^{\Delta}_{\text{ML}}$ is

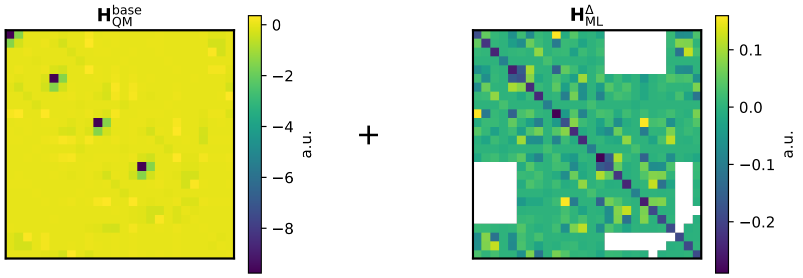

Figure S3: Illustration of the Δ-ML model idea. A baseline Fock matrix $\mathbf{H}_{QM}^{base}$ is computed with a low-level QM calculation, which can be DFT with a smaller basis (e.g., STO-3G as in our example) or a semiempirical method such as PM6. An ML model is trained to learn a Δ Fock matrix $\mathbf{H}_{ML}^{\Delta}$ that corrects the baseline so as to obtain the final Fock matrix. Long-range interactions that are not present in the ML (empty blocks in the $\mathbf{H}_{ML}^{\Delta}$ matrix shown) model are accounted for by the baseline calculation.

trained so that MO energies and charges are as close as possible to those of a higher-level, large basis QM calculation (e.g., B3LYP/def2-TZVP).

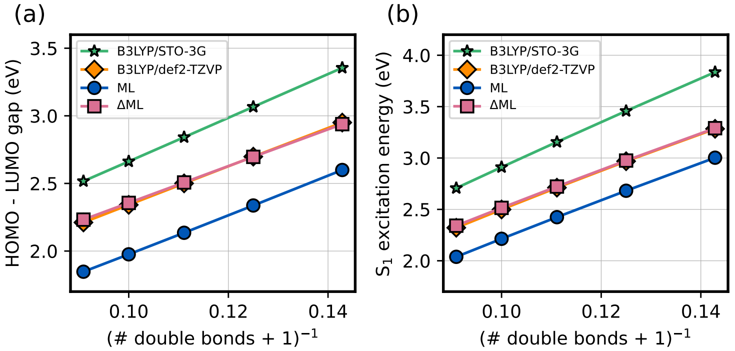

Figure S4: Comparison of the ML and Δ-ML model. Predictions on polyalkenes from 6 up to 10 double bonds. (a) Prediction of the HOMO-LUMO gap. (b) Prediction of the S_1 excitation energy.

With the Δ-ML model we achieve comparable errors on the test dataset compared to the ML model (presented in the main text). The real advantage of the Δ-ML can be seen when trying to extrapolate to much larger molecules. Figure S4 shows the predictions from the Δ-ML model as well as ML model on longer polyalkenes, for both the HOMO-LUMO gap and the excitation energy of S_1 (see also Figure 6 c and Figure S11). As we can see, we are able to get rid of the redshift that arises from the locality of the features using the Δ-ML model. Figure S5 further shows that the Δ-ML model generalizes well to very large molecules, albeit

in this case, the improvement in generalization is less striking (see Figure 6 (e) and (f)).

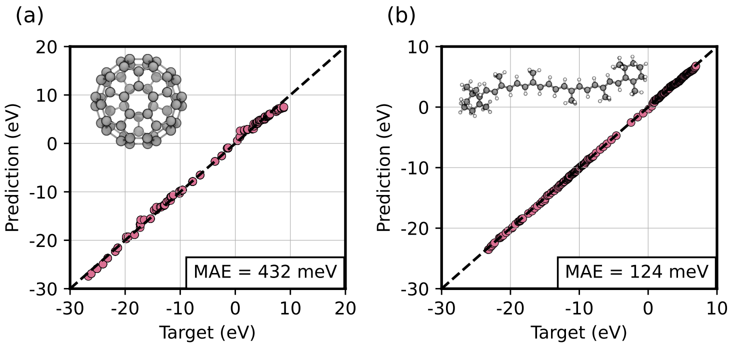

Figure S5: Δ-ML model prediction for larger molecules. The MO energies of (a) C_{60} and (b) β-carotene as predicted by the Δ-ML model. The MAE is reported in both cases, and does not include core MOs.

We stress here that other choices of the baseline Fock matrix are possible as well. For example, using a baseline obtained from a semiempirical method (e.g. PM6 or DFTB3) instead of a QM one could result in a faster Δ-ML model. This improvement in generalization comes of course at the expense of a baseline calculation, making the Δ-ML model between two and three orders of magnitude slower than the ML model (Figure 5). Nonetheless, it surely is a good starting point for the development of a Δ-ML model when a cheaper baseline is adopted.

S4 Supplementary Figures

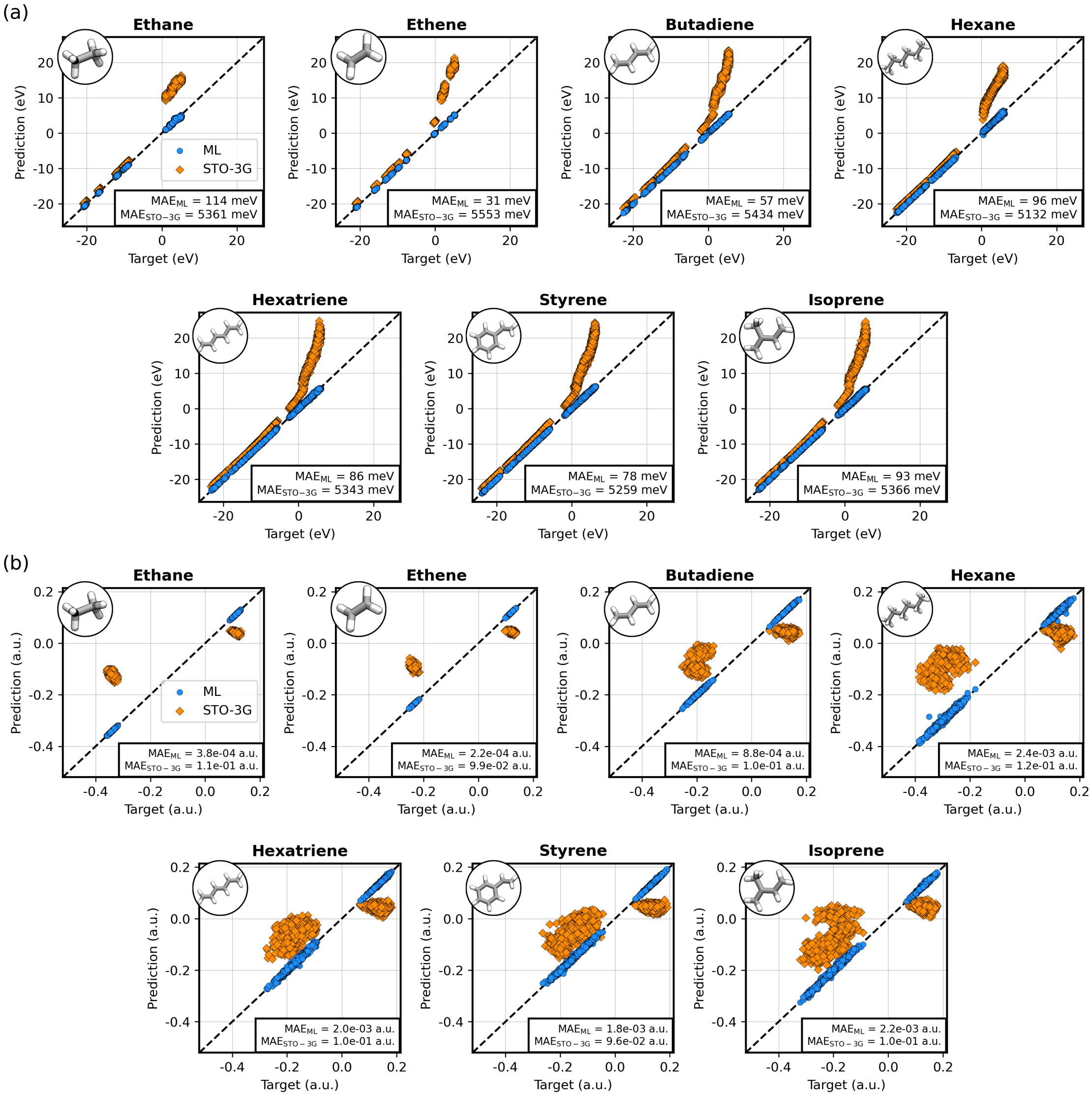

Figure S6: Performance of the LBT model on validation geometries. The target is computed with B3LYP/def2-TZVP. (a) Prediction of MO energies. Core orbitals are not shown. (b) Prediction of Löwdin atomic charges. The ML prediction is shown in blue circles. The B3LYP/STO-3G baseline is shown in orange diamonds.

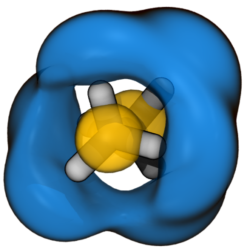

Figure S7: Visualisation of ethane's LUMO orbital. The LUMO orbital of ethane computed with B3LYP/def2-TZVP, as displayed in Figure 3 (d) presented with a reduced isodensity value instead, to emphasize its Rydberg character more effectively.

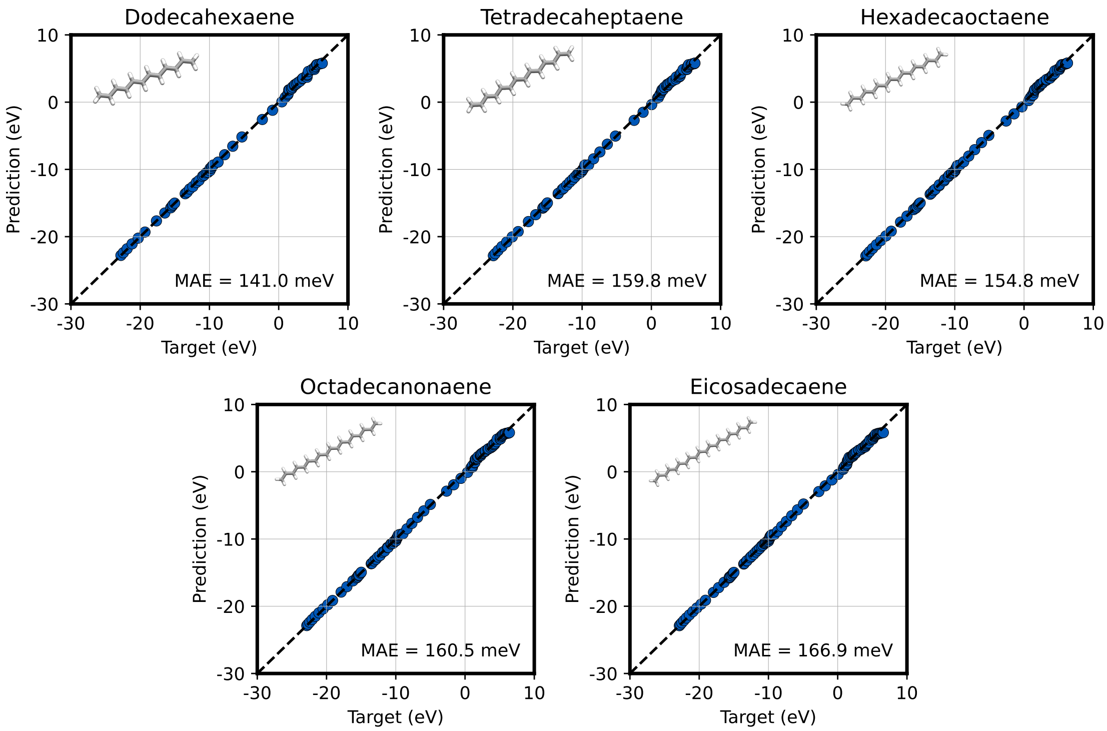

Figure S8: Prediction of the MO energy spectrum for several polyalkenes. The target is computed at B3LYP/def2-TZVP level at the optimized geometry. The energy of the core MOs is not shown. The mean absolute error (MAE) is shown for each molecule. The energy of the core MOs is not included in the MAE. Their inclusion slightly reduces the MAE.

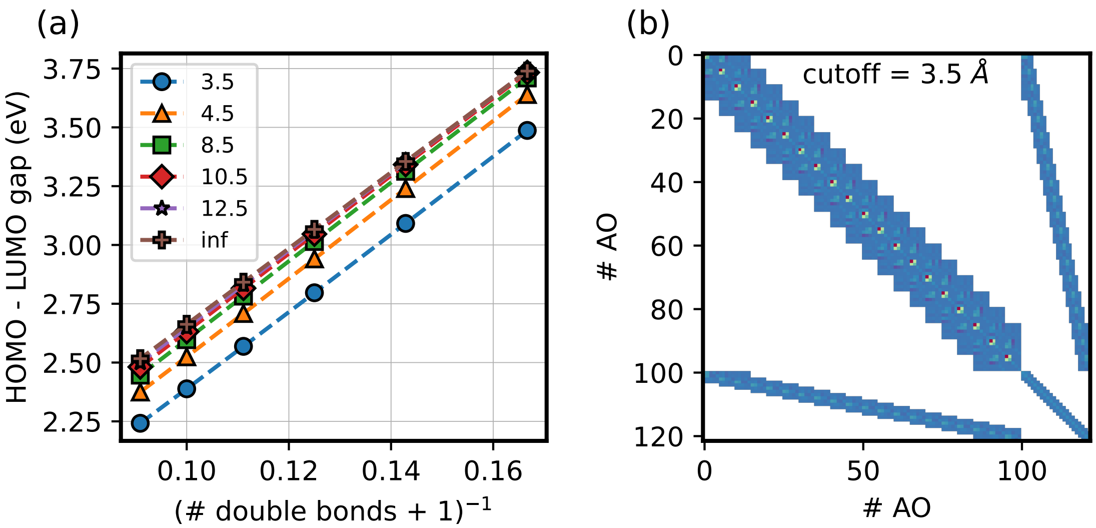

Figure S9: Effect of applying a cutoff on the elements of the Fock matrix. (a) HOMO - LUMO gap for polyalkenes from 5 up to 10 double bonds, for various cutoff values (see legend). The calculation is run at B3LYP/STO-3G level. The Fock matrix elements between two AOs whose center-to-center distance is larger than the cutoff are zeroed, the MO energies are computed, and the HOMO - LUMO gap is derived. (b) Example of a Fock matrix for eicosadecaene (10 double bonds) at its optimized geometry. A cutoff of 3.5 Å is applied. The white regions in the matrix correspond to elements that are zeroed by the application of the cutoff.

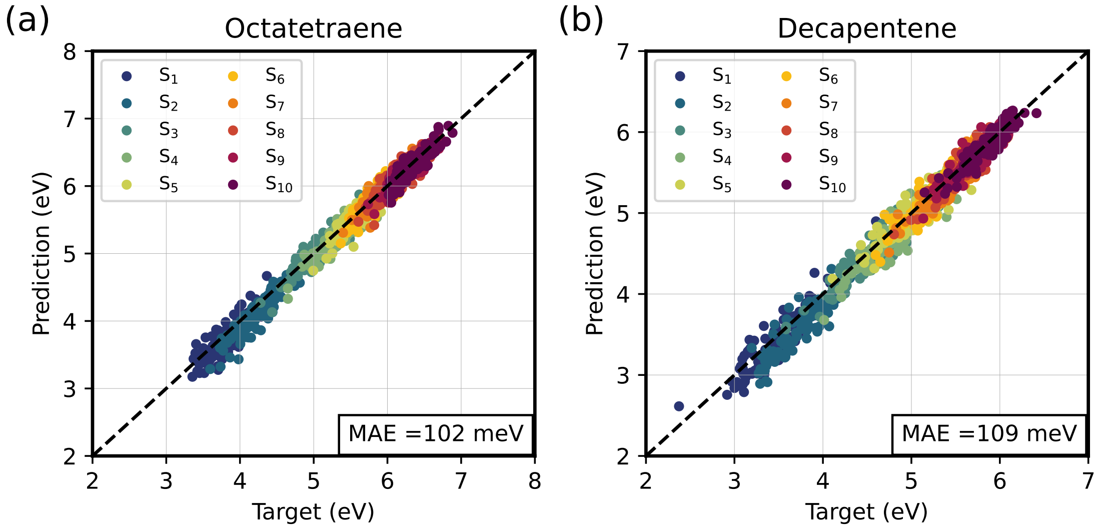

Figure S10: Prediction of the first ten singlet excited states for octatetraene and decapentene. The target is computed at the B3LYP/def2-TZVP coupled with sTDA. The prediction is that of the LBT model. The reported MAE is the absolute error averaged over all conformations and all excited states.

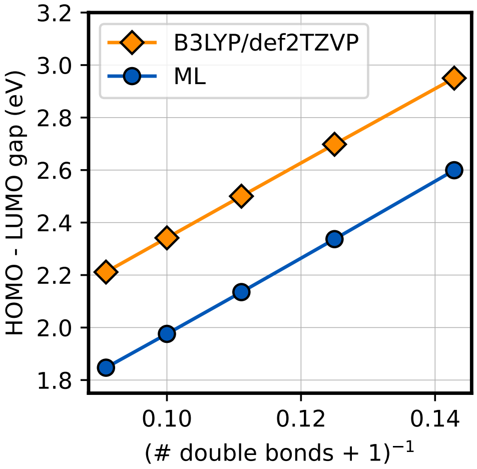

Figure S11: Target versus predicted HOMO-LUMO gaps for polyalkenes. HOMO-LUMO gap For polyalkenes, from 6 up to 10 double bonds we compare the HOMO-LUMO gap values for the target computed at B3LYP/def2-TZVP level from QM and the ones predicted by the model.

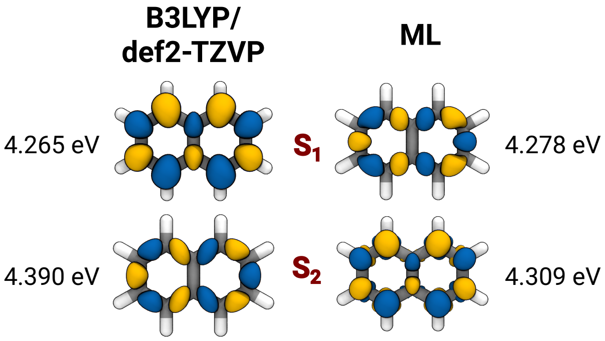

Figure S12: Excited States of Naphthalene: sTDA B3LYP/def2-TZVP vs. sTDA ML Predictions. Transition density of the first and second excited states of naphthalene, as computed with sTDA B3LYP/def2-TZVP (left) and with sTDA ML (right). The L_a state (predominant HOMO - LUMO character) is the S_1 state according to sTDA B3LYP/def2-TZVP, with a close-lying L_b state (nearly equal contributions of HOMO-1 - LUMO and HOMO - LUMO+1) about 130 meV above in energy. The order is inverted with sTDA ML, which predicts the L_b state as about 30 meV below the L_a state.

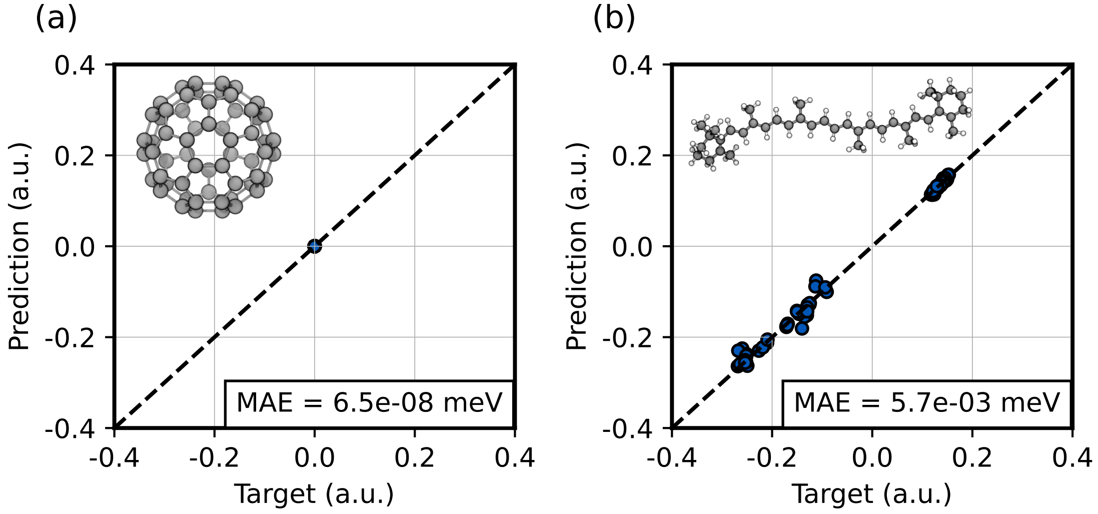

Figure S13: Predicted Löwdin charges for extrapolated molecules. Prediction of atomic Löwdin charges for (a) C_{60} and (b) β-carotene. The target charges are computed with B3LYP/def2-TZVP. The MAE is reported in both cases.

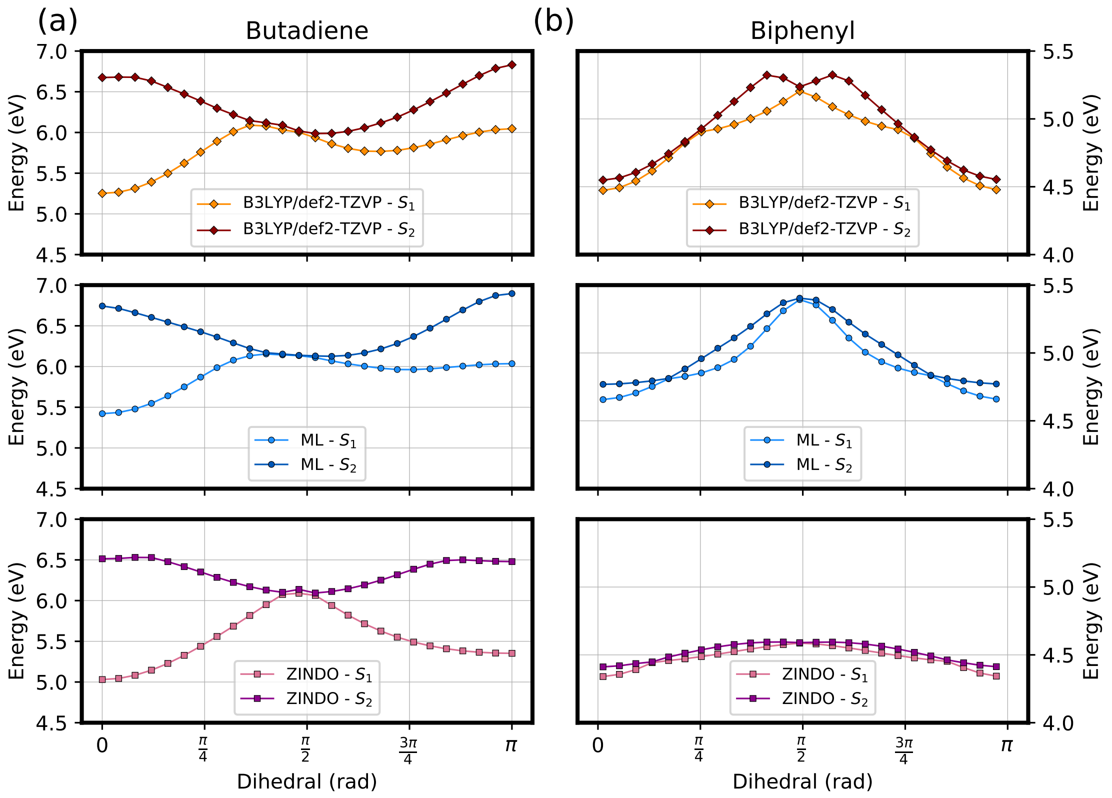

Figure S14: Comparison of the excitation energies from sTDA B3LYP/def2-TZVP, ML Model, and ZINDO. Rigid scan around the central single bond in (a) butadiene and (b) biphenyl. The excitation energies for the first two singlet excited states are reported for three different methods sTDA B3LYP/def2-TZVP (top row), the ML model (middle row), and ZINDO (bottom row).

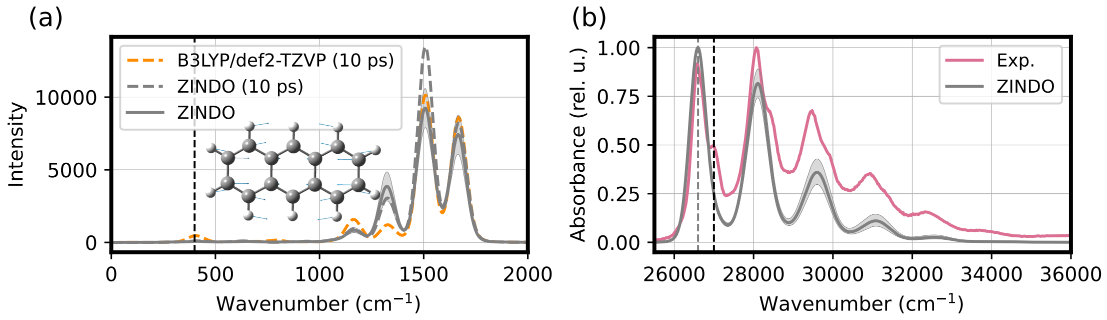

Figure S15: Spectral density and vibron spectrum. (a) Spectral density and (b) vibronic spectrum as computed with the semi-empirical method ZINDO (gray) and compared against sTDA B3LYP/def2-TZVP (orange, panel (a)) and the experimental spectrum (magenta, panel (b)). The solid line corresponds to the average over 10 ps windows along the anthracene MD. Confidence interval of 95% around the mean is reported.

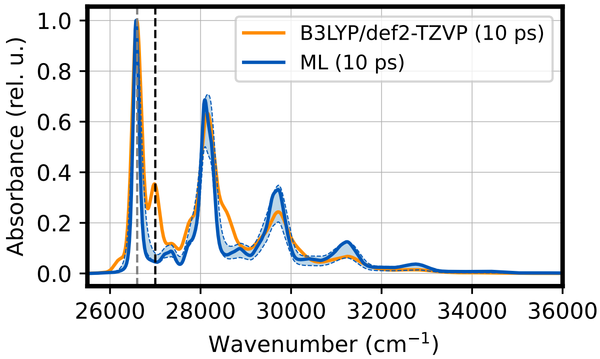

Figure S16: Vibronic spectrum of anthracene. Vibronic spectrum in a 10 ps window of the anthracene trajectory, computed with ML (blue) and with sTDA B3LYP/def2-TZVP (orange). The static disorder is not added in this spectrum, to better compare the vibronic peaks of the two spectra. The vertical dashed line denotes the peak missing from the ML prediction. The 95% confidence interval of the mean is reported for the ML model.

S5 Supplementary Tables

Table S1: Comparison of the performance of a model trained on targets computed with the minimal-basis B3LYP/STO-3G (second and third column) and the LBT model, which targets B3LYP/def2-TZVP (fourth and fifth column). We report the mean absolute errors (MAE) for both the MO energy (MAE_ϵ) and the Löwdin charges (MAE_q). The MAE_ϵ is in meV, while MAE_q is in atomic units. See also Table 1 and Figure S6.

Molecule	B3LYP/STO-3G		B3LYP/def2-TZVP	
	MAE_ϵ	MAE_q	MAE_ϵ	MAE_q
**Ethane**	16	4.1×10^{-4}	114	3.8×10^{-4}
**Ethene**	12	4.9×10^{-4}	31	2.2×10^{-4}
**Butadiene**	44	1.0×10^{-3}	57	8.8×10^{-4}
**Hexane**	63	1.7×10^{-3}	96	2.4×10^{-3}
**Hexatriene**	64	1.9×10^{-3}	86	2.0×10^{-3}
**Isoprene**	55	1.5×10^{-3}	78	1.8×10^{-3}
**Styrene**	69	2.0×10^{-3}	93	2.2×10^{-3}

Table S2: Mean absolute error (MAE) computed for the first and second singlet excited states. The MAE is computed as $N^{-1}\sum_n = 1^N |\mathcal{E}_n - \hat{\mathcal{E}}_n|$, where N is the number of samples and $\mathcal{E}_i$ is the excitation energy of the i-th data point. The MAE is reported in meV.

Molecule	ML		B3LYP/STO-3G	
	MAE_{S_1}	MAE_{S_2}	MAE_{S_1}	MAE_{S_2}
**Ethane**	45	75	8585	8513
**Ethene**	78	97	2484	2864
**Butadiene**	105	82	1455	1529
**Hexane**	188	150	5514	5538
**Hexatriene**	148	124	1123	1163
**Isoprene**	183	104	1451	1476
**Styrene**	128	178	1110	1249

Table S3: Time required to compute an excited state for the molecules from different methods. The time required to featurize a molecule is reported under "Featurization", and is absent for non-ML methods. The time required to predict (or calculate, in the case of non-ML methods) the Fock matrix is reported under "Prediction". The time required to run the sTDA is reported under "sTDA". Each time is computed on a single core of an Intel Xeon Gold 5120 Processor.

Molecule	Method	Featurization (s)	Prediction (s)	sTDA (s)	Total (s)
**Azulene**	B3LYP/def2-TZVP	-	2466.89	563.93	3030.82
	B3LYP/STO-3G	-	64.04	15.46	79.50
	ML	0.36	0.05	0.07	0.48
**Benzene**	B3LYP/def2-TZVP	-	370.86	97.22	468.08
	B3LYP/STO-3G	-	16.48	5.18	21.66
	ML	0.25	0.04	0.01	0.30
**Biphenyl**	B3LYP/def2-TZVP	-	3466.63	932.91	4399.54
	B3LYP/STO-3G	-	103.53	27.31	130.84
	ML	0.37	0.06	0.16	0.59
**Butadiene**	B3LYP/def2-TZVP	-	181.38	44.83	226.21
	B3LYP/STO-3G	-	11.51	3.27	14.78
	ML	0.22	0.04	0.01	0.27
**Dodecahexaene**	B3LYP/def2-TZVP	-	3837.90	883.43	4721.33
	B3LYP/STO-3G	-	132.11	32.29	164.40
	ML	0.33	0.05	0.23	0.61
**Eicosadecaene**	B3LYP/def2-TZVP	-	13023.30	2861.36	15884.66
	B3LYP/STO-3G	-	418.76	97.83	516.59
	ML	0.48	0.07	3.60	4.15
**Hexane**	B3LYP/def2-TZVP	-	1100.52	288.16	1388.68
	B3LYP/STO-3G	-	38.13	11.96	50.09
	ML	0.37	0.04	0.02	0.43
**Hexatriene**	B3LYP/def2-TZVP	-	487.39	119.38	606.77
	B3LYP/STO-3G	-	28.90	6.75	35.65
	ML	0.24	0.04	0.02	0.30
**Ethane**	B3LYP/def2-TZVP	-	56.57	15.03	71.60
	B3LYP/STO-3G	-	4.55	1.37	5.92
	ML	0.21	0.03	0.01	0.25
**Ethene**	B3LYP/def2-TZVP	-	29.73	8.70	38.43
	B3LYP/STO-3G	-	2.94	0.85	3.79
	ML	0.20	0.03	0.00	0.23
**Hexadecaoctaene**	B3LYP/def2-TZVP	-	7374.33	1707.01	9081.34
	B3LYP/STO-3G	-	256.04	58.11	314.15
	ML	0.39	0.06	1.00	1.45
**Isoprene**	B3LYP/def2-TZVP	-	396.94	89.22	486.16
	B3LYP/STO-3G	-	19.49	5.07	24.56
	ML	0.25	0.04	0.01	0.30
**Naphtalene**	B3LYP/def2-TZVP	-	2136.56	568.42	2704.98
	B3LYP/STO-3G	-	53.69	15.39	69.08
	ML	0.36	0.05	0.06	0.47
**Octadecanonaene**	B3LYP/def2-TZVP	-	9621.86	2238.88	11860.74
	B3LYP/STO-3G	-	327.18	74.85	402.03
	ML	0.44	0.06	1.92	2.42
**Octatetraene**	B3LYP/def2-TZVP	-	1345.15	326.00	1671.15
	B3LYP/STO-3G	-	50.00	13.18	63.18
	ML	0.27	0.04	0.04	0.35
**Styrene**	B3LYP/def2-TZVP	-	1447.99	329.12	1777.11
	B3LYP/STO-3G	-	39.86	10.40	50.26
	ML	0.29	0.05	0.03	0.37
**Tetradecaheptaene**	B3LYP/def2-TZVP	-	5507.33	1269.84	6777.17
	B3LYP/STO-3G	-	195.91	44.48	240.39
	ML	0.36	0.05	0.48	0.89

References

(S1) Nigam, J.; Willatt, M. J.; Ceriotti, M. Equivariant Representations for Molecular Hamiltonians and N -Center Atomic-Scale Properties. *The Journal of Chemical Physics* **2022**, *156*, 014115.

(S2) Nigam, J.; Pozdnyakov, S.; Ceriotti, M. Recursive Evaluation and Iterative Contraction of N -Body Equivariant Features. *The Journal of Chemical Physics* **2020**, *153*, 121101.

(S3) Nigam, J.; Pozdnyakov, S.; Fraux, G.; Ceriotti, M. Unified Theory of Atom-Centered Representations and Message-Passing Machine-Learning Schemes. *The Journal of Chemical Physics* **2022**, *156*, 204115.

(S4) Musil, F.; Veit, M.; Goscinski, A.; Fraux, G.; Willatt, M. J.; Stricker, M.; Ceriotti, M. Efficient Implementation of Atom-Density Representations. *The Journal of Chemical Physics* **2021**, *154*, 114109.

(S5) Yue, S.; Muniz, M. C.; Andrade, M. F. C.; Zhang, L.; Car, R.; Panagiotopoulos, A. Z. When do short-range atomistic machine-learning models fall short? *The Journal of Chemical Physics* **2021**, *154*.

(S6) Anstine, D. M.; Isayev, O. Machine Learning Interatomic Potentials and Long-Range Physics. *The Journal of Physical Chemistry A* **2023**, *127*, 2417–2431.

(S7) Kabylda, A.; Vassilev-Galindo, V.; Chmiela, S.; Poltavsky, I.; Tkatchenko, A. Efficient interatomic descriptors for accurate machine learning force fields of extended molecules. *Nature Communications* **2023**, *14*.

(S8) Grisafi, A.; Ceriotti, M. Incorporating Long-Range Physics in Atomic-Scale Machine Learning. *The Journal of Chemical Physics* **2019**, *151*, 204105.

(S9) Unke, O.; Bogojeski, M.; Gastegger, M.; Geiger, M.; Smidt, T.; Müller, K.-R. SE (3)-

Equivariant Prediction of Molecular Wavefunctions and Electronic Densities. *Advances in Neural Information Processing Systems* **2021**, *34*.
