# Supplementary material for: Electronic Excited States from Physically Constrained Machine Learning
Source: ACS Cent Sci. 2024 Feb 29;10(3):637–48. doi: 10.1021/acscentsci.3c01480 (PMC10979507; doi:10.1021/acscentsci.3c01480)
Supplement: Supplementary file 2 — oc3c01480_si_002.pdf [file oc3c01480_si_002.pdf]

oc-2023-01480s.R1

Name: Peer Review Information for "Electronic excited states from physically-constrained machine learning"

First Round of Reviewer Comments

Reviewer: 1

Comments to the Author

The authors introduce a machine learning (ML) strategy that innovatively combines data-driven modeling and quantum mechanical (QM) calculations. The core of their ML model incorporates an intermediate layer emulating a minimal basis single-particle electronic Hamiltonian. This layer is instrumental in computing the energies of molecular orbitals (MO) and atomic charges. Through training the models based on MO energies obtained at a larger basis, remarkably accurate predictions are achieved.

The article succinctly elucidates the methodology for predicting properties beyond MO energies and atomic charges, especially for larger molecules not included in the training set. The authors showcase the model's capability in predicting excitation energies and highlight its transferability by successfully predicting properties for previously unseen and larger molecules. While this contribution underscores the advantages of the hybrid ML solution, it also addresses potential disadvantages. I recommend the publication of this article with the minor comments below addressed.

Q1: The authors have convincingly demonstrated that combining MO energies and atomic charges as targets enhances the model's balance and improves predictions compared to direct Hamiltonian learning. Could the authors elaborate on the tradeoffs incorporated into the combined loss function for these two properties?

Q2: The paragraph on page 10, starting at line 36, is challenging to follow. To enhance clarity, I suggest revising the text to provide better guidance to the reader. Consider incorporating specific references to Figure 3 or presenting a comparative figure illustrating errors in large-basis targets versus those targeting properties computed with a minimal basis. This adjustment would enhance the reader's comprehension of the discussed content.

Reviewer: 2

Comments to the Author

The manuscript entitled "Electronic excited states from physically-constrained machine learning" by Edoardo Cignoni, Divya Suman, et al. presents a novel approach to developing a single-particle machine learning (ML)-based single-particle Hamiltonian. The novelty is to create an ML-Hamiltonian similar in size to a minimal basis Hamiltonian, but targeting molecular orbital energies and Löwdin charges derived from a triple zeta calculation. The authors use equivariant representations to preserve the symmetry inherent in the Hamiltonian. Furthermore, they demonstrate the applicability of the ML Hamiltonian in predicting molecular orbitals and Löwdin charges for larger systems by utilizing training on seven hydrocarbons and different conformations. The article explores the calculation of excited state energies and demonstrates their usefulness in the simplified Tamm-Dancoff approximation (sTDA). In addition, the vibronic spectrum of azulene is calculated. The conceptual framework behind the generation of a minimal-size surrogate Hamiltonian for accurate data evaluation is indeed commendable and innovative.

However, the study reaches its limits in terms of applicability beyond the training set, especially when comparing to methods such as the semiempirical ZINDO method for vibronic spectra or excited states of molecules that were not included in the original training. The limited performance evaluation, which is restricted to the first and second excited singlet states, is a concern in addition to the technical limitations (cutoff) that affect scalability for larger systems.

For a more comprehensive evaluation, several questions emerge:

1) The model's treatment of differently sized molecules within the training set and adaptations required for incorporating other atom types need elucidation. Is the model readily applicable to diverse datasets such as QM7-X, or would substantial modifications be necessary? Have the authors explored other datasets akin to QM7-X?

2) Understanding the limitations on system size is crucial. What's the upper threshold for system size inclusion in the training set without compromising efficient training and prediction? Can the authors provide insights into this aspect?

3) The evaluation of solely the first and second excited singlet states within the training set raises queries about neglecting higher electronic states. Additionally, when predicting excited states for molecules outside the training set, exploring more than just the first excited state could enhance the study's depth.

4) The almost constant value of Löwdin charges around 0 for the prediction of C60 prompts inquiry into whether this stems from a limitation within the model for such systems. Can the authors elucidate further on this point?

5) The references are missing in the supporting information.

Author's Response to Peer Review Comments:

STI – IMX - COSMO
Laboratory of Computational Science and Modelling

Lausanne, January 16, 2024

Dear Editor,

We thank the anonymous Reviewers for the time they dedicated to reviewing our manuscript and for their overall positive evaluation. Please find attached a point-by-point reply to the queries and issues raised by Reviewer 2. The main changes involve the inclusion of ten excited states in our benchmarks, and a more explicit discussion of the scaling of the method with chemical complexity and system size. We did also perform some demonstrative calculations on molecules containing more chemical elements, but we decided to include them only in this response, as they are not up to the same standard as the main calculations we had performed, and do not - in our opinion - add much to the core message of this work.
We have also implemented the changes requested by the Editorial Team, and corrected a few typos and minor lexical errors. The changes to the main text are highlighted in blue in the annotated copy of our revised manuscript.

We hope to have satisfactorily responded to the reviewers' questions, and to have elevated the manuscript to the level that is needed for publication in ACS Central Science.

Thank you for your consideration.

Yours sincerely,

Michele Ceriotti
on behalf of all Authors

EPFL STI-IMX-COSMO
Pr. Michele Ceriotti
MXG 340, Station N° 12

E-mail: michele.ceriotti@epfl.ch

Reviewer: 1

Recommendation: Publish in ACS Central Science after minor revisions noted.

Comments:

The authors introduce a machine learning (ML) strategy that innovatively combines data-driven modeling and quantum mechanical (QM) calculations. The core of their ML model incorporates an intermediate layer emulating a minimal basis single-particle electronic Hamiltonian. This layer is instrumental in computing the energies of molecular orbitals (MO) and atomic charges. Through training the models based on MO energies obtained at a larger basis, remarkably accurate predictions are achieved.

The article succinctly elucidates the methodology for predicting properties beyond MO energies and atomic charges, especially for larger molecules not included in the training set. The authors showcase the model's capability in predicting excitation energies and highlight its transferability by successfully predicting properties for previously unseen and larger molecules. While this contribution underscores the advantages of the hybrid ML solution, it also addresses potential disadvantages. I recommend the publication of this article with the minor comments below addressed.

Authors' reply: We sincerely thank the reviewer for the positive comments.

Q1: The authors have convincingly demonstrated that combining MO energies and atomic charges as targets enhances the model's balance and improves predictions compared to direct Hamiltonian learning. Could the authors elaborate on the tradeoffs incorporated into the combined loss function for these two properties?

Authors' reply: Indeed, the loss over the MO energies and the loss over the atomic charges enter the combined loss with different weights, so that the two components have approximately the same weight in the total loss. In this way, training with backpropagation ensures that the model is not imbalanced towards one of the two properties of the electronic Hamiltonian. We have found this essential to prevent overfitting and to generalize well to diverse and larger molecules. As the reviewer suggests, having the two losses combined also means obtaining a model that achieves the best compromise in accuracy between the MO energies and the atomic charges. If the aim of the model is just to provide accurate MO energies, for example, setting the weight of the loss over atomic charges to zero will provide more accurate MO energies for molecules included in the training set (as is shown in Table 1 of the main text). However, we observe that such a model, being severely underdetermined, has a strong tendency to overfit. Its limits become apparent when predicting other properties (such as atomic charges). This tradeoff between the accuracy on MO energies and atomic charges is also beneficial for the generalization quality of the model. Following the reviewer's advice, we have added the following sentence to the text to better stress this point, at the end of Section 2.1:

"Combining multiple targets […] and atomic charges. The MO energies have slightly larger errors (Table 1), as the composite loss forces the model to both reproduce the MO energies and the overall electronic density (via the atomic charges). Model 3 thus more faithfully respects the underlying physics, which helps when generalizing to new properties and molecules, as we show in the following sections."

Q2: The paragraph on page 10, starting at line 36, is challenging to follow. To enhance clarity, I suggest revising the text to provide better guidance to the reader. Consider incorporating specific references to

Figure 3 or presenting a comparative figure illustrating errors in large-basis targets versus those targeting properties computed with a minimal basis. This adjustment would enhance the reader's comprehension of the discussed content.

Authors' reply: We agree with the reviewer that the paragraph can be rewritten so that it will no longer result in a heavy read. We have reframed the paragraph as follows, also adding references to the figure pointed out by the reviewer and adding another table in the SI (now Table S1) where the performance of the minimal-basis and large-basis models is compared:

"Compared to a minimal-basis prediction (see Table 1), the errors of a model targeting a large-basis Hamiltonian (LBT model) are considerably larger (i.e., up to a factor of 10 for ethane, see Figure S6 and Table S1). The larger inaccuracy of the LBT model is to be expected, as the target is far more complex than in the minimal-basis case. However, these errors are at least an order of magnitude smaller than the error of an explicit QM calculation in a minimal basis (see Figure 3 (b) and (c)). Indeed, the average MAE on MO energies over the test conformations is 78 meV for the LBT model, to be compared with the 5250 meV for the explicit minimal-basis QM calculation (Figure 3 (b)). This shows that the LBT model learns an effective pseudo-Hamiltonian that reproduces the desired electronic properties of an expensive QM calculation in a large basis to a high accuracy.

Thanks to the prediction of the electronic Hamiltonian, the LBT model retains some of the interpretability of a QM calculation. Indeed, it is possible to visualize the MO shapes to understand the quality of the prediction, as we show in Figure 3 (d) for various molecules. We stress in passing that even though the pseudo-Hamiltonian has the symmetries of a minimal basis H, it is not explicitly tied to a choice of atom-centered functions, and any basis with the correct symmetry can be used to visualize the MOs. In Figure 3 (d), we choose the STO-3G basis for simplicity. The LUMO orbital of ethane is the only one exhibiting a mismatch with the ML prediction. The difficulty in predicting this MO lies in its Rydberg character (see also Figure S7). Rydberg orbitals are known to be present for calculations of small molecules in the gas phase, especially when using atomic bases with diffuse orbitals. Their diffused character appears to be particularly challenging for the LBT model. All the other orbitals show that the symmetry and nodal structure of the LBT one-electron wavefunctions are learned correctly (see Figure 3 (d)). For the remainder of this study, we will focus exclusively on this type of LBT hybrid models that offer an excellent trade-off between accuracy and computational expense."

Additional Questions:

Quality of experimental data, technical rigor: Top 1%

Significance to chemistry researchers in this and related fields: Top 1%

Broad interest to other researchers: Top 1%

Novelty: Top 1%

Is this research study suitable for media coverage or a First Reactions (a News & Views piece in the journal)?: Yes

Reviewer: 2

Recommendation: Reconsider after major revisions noted.

Comments:

The manuscript entitled "Electronic excited states from physically-constrained machine learning" by Edoardo Cignoni, Divya Suman, et al. presents a novel approach to developing a single-particle machine learning (ML)-based single-particle Hamiltonian. The novelty is to create an ML-Hamiltonian similar in size to a minimal basis Hamiltonian, but targeting molecular orbital energies and Löwdin charges derived from a triple zeta calculation. The authors use equivariant representations to preserve the symmetry inherent in the Hamiltonian. Furthermore, they demonstrate the applicability of the ML Hamiltonian in predicting molecular orbitals and Löwdin charges for larger systems by utilizing training on seven hydrocarbons and different conformations. The article explores the calculation of excited state energies and demonstrates their usefulness in the simplified Tamm-Dancoff approximation (sTDA). In addition, the vibronic spectrum of azulene is calculated. The conceptual framework behind the generation of a minimal-size surrogate Hamiltonian for accurate data evaluation is indeed commendable and innovative.

Authors' reply: We sincerely thank the reviewer for the positive comments.

However, the study reaches its limits in terms of applicability beyond the training set, especially when comparing to methods such as the semiempirical ZINDO method for vibronic spectra or excited states of molecules that were not included in the original training. The limited performance evaluation, which is restricted to the first and second excited singlet states, is a concern in addition to the technical limitations (cutoff) that affect scalability for larger systems.

Authors' reply: While the *implementation* of the method has certainly its limits - in particular due to the fact it does not (yet) allow for distributed training over multiple computer nodes, that restricts somewhat the maximum size of the training set - the methodology can be extended without much conceptual difficulty. For instance, as we discuss below, it is certainly possible to predict more than two excited states, and as we show in the SI the reason not to increase the cutoff distance is that (for the training set size that we use) doing so would result in an overall degradation of performance, not that it is challenging on a conceptual or practical level. Extension to a more chemically diverse dataset is also underway.

For a more comprehensive evaluation, several questions emerge:

1) The model's treatment of differently sized molecules within the training set and adaptations required for incorporating other atom types need elucidation. Is the model readily applicable to diverse datasets such as QM7-X, or would substantial modifications be necessary? Have the authors explored other datasets akin to QM7-X?

Authors' reply: We agree with the reviewer that we should explain better to the reader whether the model is applicable straightforwardly to other atom types, besides the C and H analyzed in the manuscript. Indeed, our model can treat arbitrary atom types, and is by no means restricted to hydrocarbons. In fact, we want to stress that the features and regressors at the basis of our model are precisely the same as those used in Ref. [1]. In that work, some of us showed the applicability of the model to the QM7-CHNO dataset. Ultimately, every type of atom can be accounted for by our model because it will be described by a dedicated set of features that are uniquely defined for that atom. Therefore, there is no theoretical limitation on the applicability of our framework to atoms different from C and H, and no substantial modifications to the

model are required to train on different datasets. To stress this important point further, we have added the following sentence to the main manuscript, at the end of the first paragraph of the "Results" section:

"We finally stress that our methodology is by no means limited to just hydrocarbons, and can be extended straightforwardly to more diverse datasets."

We have not yet tackled the challenge of training on datasets such as QM7-X, as those datasets are very large, posing substantial challenges both for the training (as we discuss above and in the following answer, we do not have yet a distributed training implementation) and for the evaluation of the training set (the public QM7-X does not contain Hamiltonian data, and the QM calculations to compute the B3LYP/def2-TZVP Fock matrices would require a dedicated allocation on a large computing platform). To show that our model is indeed applicable to other datasets and atom types, we have performed a demonstrative training exercise of indirect learning on 200 structures of the QM7 dataset, minimizing the combined loss on MO energies and atomic charges and computing the target with B3LYP/STO-3G. That is, our training exercise is limited to a minimal-basis target, as computing the target with a triple-zeta basis is way more expensive. The structures that we have selected comprise C, H, N, and O atoms. We have used 150 structures to train the model, and 50 structures to compute the test errors. As shown in the table below, we obtain a test error on MO energies of 71.2 meV from the indirect model that uses the combined loss function on eigenvalues and Löwdin charges ($\mathcal{L} = \mathrm{MSE}_{\varepsilon,q}$), which compared to the 330 meV obtained with the "direct" model ($\mathcal{L} = \mathrm{MSE}_{\mathbf{H}}$) of Ref. [1] confirms the rough order of magnitude improvement already reported in Table 1 of the main manuscript. We also show here the parity plots for the valence MO energies for the two different models. Different colors on the plot correspond to the different molecules of the test set. We are then confident that training on a triple-zeta target (LBT model) would yield an error that reflects the one reported in Table 1 (i.e., intermediate between 70 meV and 330 meV). To provide an idea of the training time, a single epoch over the train dataset (150 unique molecules) required about 19 s, and it took between one and two days to converge the model. We are currently working on improving the implementation of our method, and in the near future we expect to be able to demonstrate applications that require the kind of diverse chemical composition and structural distortion that is typical of datasets such as QM7-X. .

Loss function	$\mathrm{RMSE}_{\varepsilon}$ (meV)	RMSE_{q} (A.U)
$\mathcal{L} = \mathrm{MSE}_{\mathbf{H}}$	330	9.7×10^{-3}
$\mathcal{L} = \mathrm{MSE}_{\varepsilon,q}$	71.2	3.6×10^{-3}

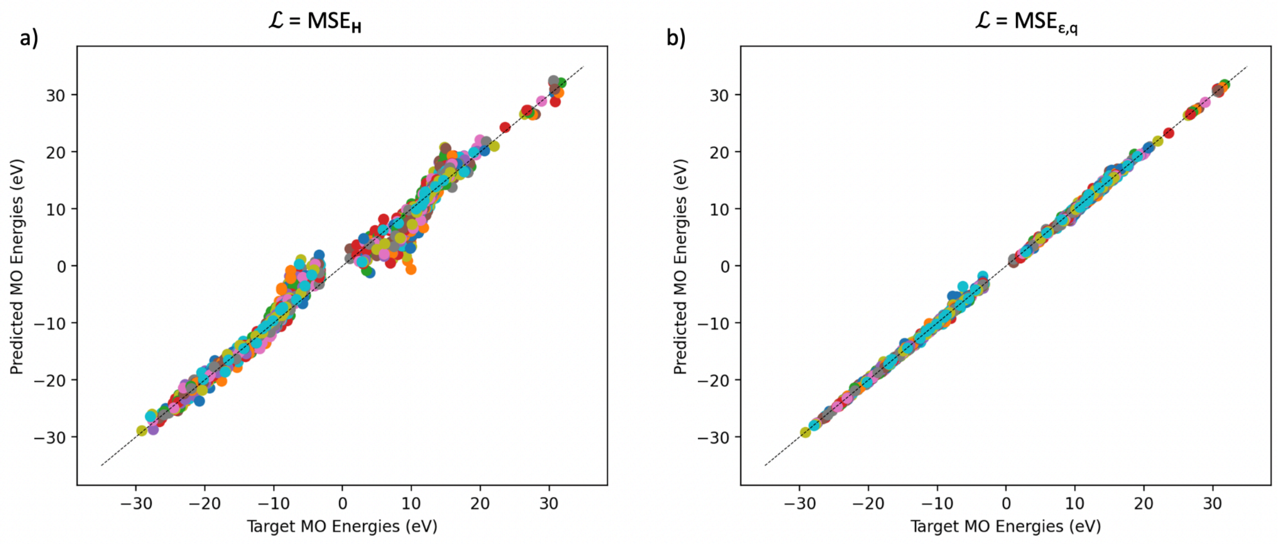

[1] Nigam, Jigyasa, Michael J. Willatt, and Michele Ceriotti. "Equivariant representations for molecular Hamiltonians and N-center atomic-scale properties." The Journal of Chemical Physics 156.1 (2022).

2) Understanding the limitations on system size is crucial. What's the upper threshold for system size inclusion in the training set without compromising efficient training and prediction? Can the authors provide insights into this aspect?

Authors' reply: We agree with the reviewer that indeed understanding the limitations on system size is crucial. In this respect, one needs to distinguish between the formal scaling of the methodology, and the limitations introduced by the implementation, that - as we discussed already - is meant as a proof of principle and not as a final release. To provide a direct answer to the reviewer's queries, we provide here some information about the memory requirements and the speed bottlenecks of our procedure.

For what concerns the prediction phase, the time-demanding steps are the calculation of the features and the prediction itself. Features are associated with orbital pairs, and so would formally scale with the square of the size of the molecule. Given however that features have a finite range, matrix elements between far-apart atoms can be ignored, and so the asymptotic scaling is linear. To give an idea of the time required for computing the features, besides the information already reported in Table S3, it requires about 300 ms for stilbene, 2.9 s for β-carotene, and 5.36 s for C_{60} on a simple workstation and a single core. Once the features are calculated, it requires about 65 ms for stilbene, 145 ms for β-carotene, and 174 ms for C_{60} to predict the Fock matrix, diagonalize it to obtain the MO energies and coefficients, and compute the Lowdin charges. Even though matrix diagonalization, needed in the indirect model to estimate eigenvalues, has a cubic scaling with the matrix size, this cost would not become relevant until much larger structures are considered; the use of a minimal-basis ansatz reduces the prefactor greatly, and ultimately one may resort to linear-scaling algorithms to estimate the most relevant part of the spectrum. Given that both feature calculation and model predictions are orders of magnitude faster than the corresponding DFT B3LYP/def2-TZVP calculation, the current code can already scale efficiently to large molecules such as β-carotene and C_{60}.

For what concerns the training time, the current code is more limited. At present, it takes one or two days to train on 1750 conformations of the molecules reported in the train set (250 conformations per

molecule). The time for completing one epoch is around 9.5 seconds. We have added this information in the Supplementary Information, where the details of the model training are provided:

"Training with these settings required about 9.5 s for one epoch, and between one and two days for the model to converge."

For a comparison, training on 500 conformations of butadiene requires about 1.8 seconds per epoch. We want to emphasize that there are implementation details that will need to be improved in the future for a high performant code. For example, a bottleneck that considerably impacts the training time is reconstructing the full Fock matrix given the prediction in terms of irreducible blocks. In fact, around 70% of the time required to predict the Fock matrix precisely involves combining the various symmetry blocks of the Fock in a single matrix, after they are predicted by the model. We have optimized parts of the code so that assigning a value to a given block over multiple frames is fast, because the operation is vectorized using PyTorch operations. Still, looping over the symmetry blocks is done with Python, which slows down the reassembling of the Fock matrix. Analogously, also the loop over different molecules is performed in Python, which means that training on many different molecules could be sped up substantially. Some ideas on making the code faster on this side would be to e.g. extend the PyTorch code with custom operations written in C++/CUDA to make the reassembling of the Fock matrix faster. We have refrained from doing so in our current version of the code, because optimizing these routines involves a considerable amount of work that we consider premature at this stage, where we are investigating the generalization and scaling properties of our strategy.

We also provide information on the memory requirements to store the features, going from small to large molecules. To measure the memory occupancy, we have expressed the features as NumPy arrays with 64-bit double precision values and obtained the size calling the `nbytes` attribute of the NumPy array.

**Molecule**	**Features (kB)**
Ethene	415
Ethane	516
Butadiene	1078
Benzene	2085
Isoprene	1671
Hexatriene	1711
Styrene	2851
Azulene	3801
Naphthalene	3766
Hexane	2241
Biphenyl	4594

Dodecahexaene	3611
Tetradecaheptaene	4245
Hexadecaoctaene	4878
Octadecanonaene	5511
Eicosadecaene	6145

A HPC node with e.g. 1 TB of RAM would then be able to store in memory 2.5 million ethane (2 heavy atoms) conformations or 174 thousand eicosadecaene molecules (10 heavy atoms). These memory requirements show that, memory-wise, it is entirely feasible to train on a lot of different conformations and molecules, avoiding the re-calculation of the features at each epoch. The prediction (Fock matrix with a minimal-basis size) is a lot less demanding, especially if one avoids storing the elements that are exactly zero due to the cutoff employed (last column of the following table):

Prediction:

**Molecule**	**Minimal-basis Fock (kB)**	**Minimal-basis Fock with cutoff, (kB)**
Ethane	2.0	2.0
Ethene	1.5	1.5
Butadiene	5.3	4.5
Benzene	10.1	9.5
Isoprene	8.5	7.3
Hexatriene	11.3	7.3
Styrene	18.0	13.1
Azulene	26.3	17.9
Naphthalene	26.3	17.7
Hexane	15.1	9.8
Biphenyl	38.3	21.6
Dodecahexane	42.8	15.7
Tetradecaheptaene	57.8	18.5
Hexadecaoctaene	75.0	21.4

Octadecanonaene	94.5	24.2
Eicosadecaene	116.3	27.0

We conclude by stressing again even if our code has not been optimized yet, it is in principle applicable to arbitrarily complex and large molecules - even though in practice the execution might become slow, or require high-memory nodes . (for example, in the previous answer we report 19 s per epoch when training on 150 different molecules with H, C, N, O atoms). We avoided focusing on the code performance in the main manuscript, stating instead that "the current implementation is not optimized for speed, being instead focused on making it easy to test new ideas: therefore a more efficient calculation of symmetry-adapted features, as well as the use of more sophisticated model architectures, leave much room to further improve accuracy and computational requirements of the ML model", to avoid giving the impression that our code can already be used straightforwardly in arbitrary scenarios.

3) The evaluation of solely the first and second excited singlet states within the training set raises queries about neglecting higher electronic states. Additionally, when predicting excited states for molecules outside the training set, exploring more than just the first excited state could enhance the study's depth.

Authors' reply: We thank the reviewer for raising this point, we indeed agree that expanding the prediction to more excited states will be beneficial for the quality of the work. Indeed, a known point of difficulty when modelling the excited states of molecules with machine learning is achieving a balanced description of many excited states [1], i.e., predicting many excited states without incurring in a dramatic increase in prediction error. Following the reviewer's advice, we have expanded the prediction to the first 10 excited states, for both the molecules already included in the training set, and, crucially, for molecules that were not included in the train data. We have obtained a well-balanced description for the molecules in the training set (predicting on test conformations):

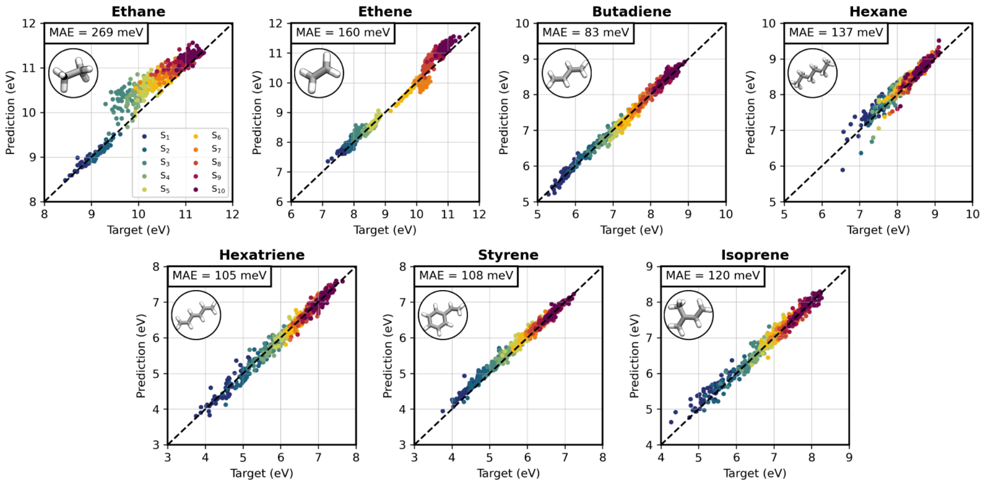

Here, the larger errors witnessed for Ethane and Ethene can be traced to the presence of Rydberg orbitals for such small molecules, as we have already evidenced in the manuscript. The excited states for all the other molecules are predicted with a remarkable accuracy, and this remains true when predicting for molecules outside the train set, like for octatetraene and decapentene:

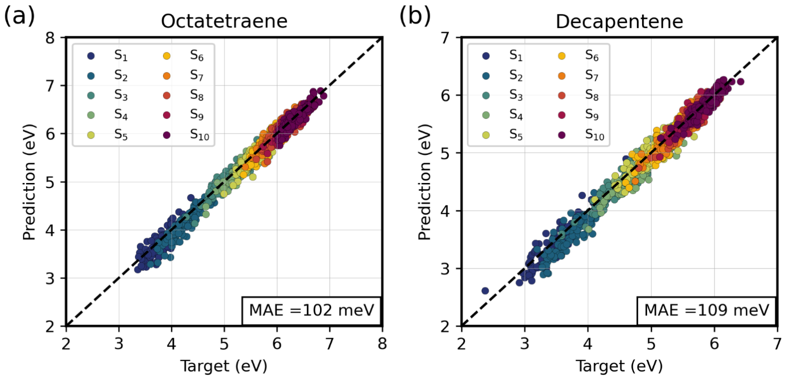

We have also predicted the first three excited states for the polyalkenes and aromatic molecules, therefore expanding Figure 6 of the manuscript:

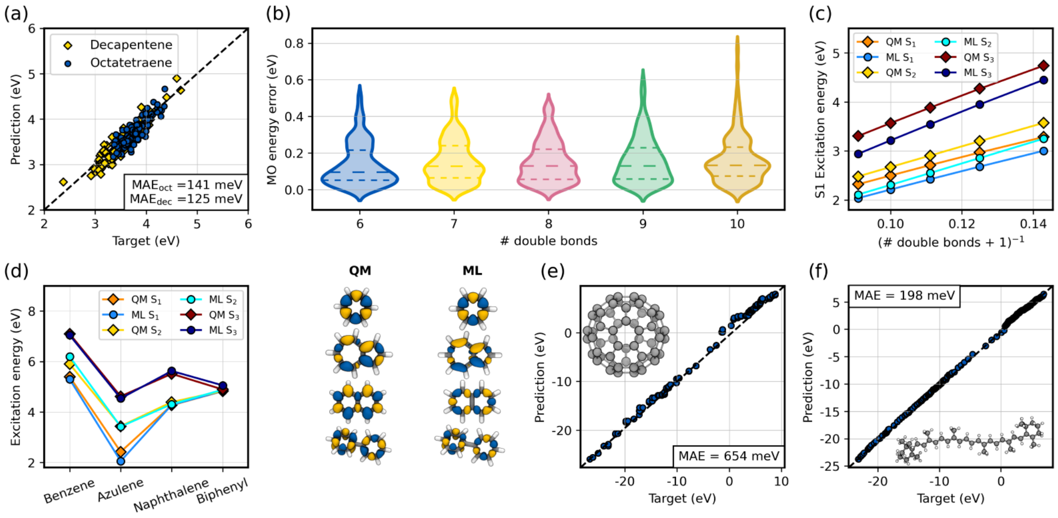

For the case of polyalkenes and aromatic molecules we witness what was already visible for the first excited state, namely that there is a systematic red shift for the optimized structure of polyalkenes, and that the excitation energies for aromatic molecules are well-predicted. We have incorporated these figures in the manuscript (the first and third are in the main text, while the prediction for octatetraene and decapentaene is in the SI). Since two figures of the main manuscript have been changed due to these new results, the discussion of the section "Electronic excitations from an ML Hamiltonian" and "Extrapolative predictions" have been modified in multiple parts. In particular, we have again noted that the presence of Rydberg orbitals for small molecules deteriorates the prediction:

"We also note that the prediction for Ethane and Ethene, the smallest molecules in the training set, suffer from the presence of Rydberg orbitals that we have previously evidenced."

And remarked that our strategy, namely predicting excited states by first learning a more fundamental molecular quantity (the molecular Hamiltonian), is a viable way for obtaining a balanced description of multiple excited states at once:

"Furthermore, as obtaining a balanced description of many excited states with ML is known to be challenging,[10] our results show that learning a molecular Hamiltonian is a viable way for obtaining a consistent prediction of excited states."

Finally, we refer the reader to Figure S10 in the SI, which shows the prediction of the first ten excited states for octatetraene and decapentaene:

"Indeed, Figure S10 shows that similar levels of accuracy can be achieved up to the tenth singlet excited state does not incur a decrease in the accuracy."

We believe that these new results considerably improve the manuscript, and indeed they evidence the ability of our model to consistently predict many excited states, a feature that we did not emphasize in our initial presentation.

[1] Dral, Pavlo O., and Mario Barbatti. "Molecular excited states through a machine learning lens." Nature Reviews Chemistry 5.6 (2021): 388-405.

4) The almost constant value of Löwdin charges around 0 for the prediction of C60 prompts inquiry into whether this stems from a limitation within the model for such systems. Can the authors elucidate further on this point?

Authors' reply: We are of the opinion that the almost-constant zero atomic charge for C_{60} is just a consequence of the symmetry of the molecule. Indeed, in our calculations we have employed a highly symmetric structure (I_h) where all atoms are equivalent by symmetry, and therefore any deviation from charge neutrality is a manifestation of numerical noise. We also note that the prediction matches well the QM calculation, since both the x and y axes in Figure S12 are multiplied by 10^{-6}. These charges thus approach the maximum precision of a 32-bit single-precision float variable, and indeed the small fluctuations observed could be the consequence of some truncation at single-precision level of intermediate quantities in the calculation. Recomputing the Lowdin charges in a QM calculation with Gaussian 16 at the B3LYP/def2-TZVP level yields Lowdin atomic charges that are exactly zero up to the sixth decimal digit. This level of detail is more than enough for all practical scenarios where a researcher would want to use these atomic charges for interpretation or for further calculations. Given all these considerations, we believe that there is no intrinsic limitation in our model for such a system. However, we do understand the concerns of the reviewer regarding the parity plot in Figure S12 (a), which we attribute to an unfortunate choice of the axes scale. We have rescaled both axes to a range that is more "natural" for an atomic charge (e.g., from –0.4 to 0.4) and simplified the caption in Figure S12.

(a)

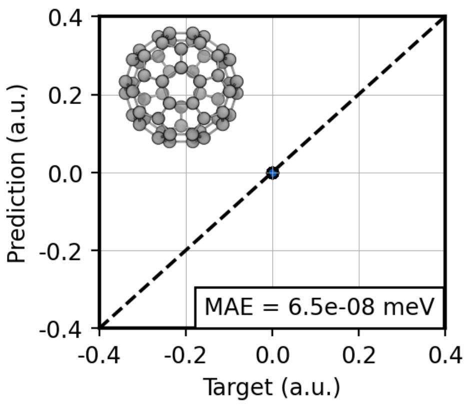

Displayed in this way, we believe it is clear that the model prediction matches, up to numerical precision, the zero atomic charge that is consistent with the molecular symmetry of C_{60}.

5) The references are missing in the supporting information.

Authors' reply: We do apologize for the inconvenience; we have restored the references in the Supporting Information file.

Additional Questions:

Quality of experimental data, technical rigor: High

Significance to chemistry researchers in this and related fields: Top 5%

Broad interest to other researchers: Moderate

Novelty: Top 5%

Is this research study suitable for media coverage or a First Reactions (a News & Views piece in the journal)?: No
